# Different recovery patterns of CMV-specific and WT1-specific T cells in patients with acute myeloid leukemia undergoing allogeneic hematopoietic cell transplantation: Impact of CMV infection and leukemia relapse

**DOI:** 10.3389/fimmu.2022.1027593

**Published:** 2023-02-07

**Authors:** Xiao-Hua Luo, Thomas Poiret, Zhenjiang Liu, Qingda Meng, Anurupa Nagchowdhury, Per Ljungman

**Affiliations:** ^1^ Department of Hematology, The First Affiliated Hospital of Chongqing Medical University, Chongqing, China; ^2^ Department of Laboratory Medicine, Karolinska Institutet, Stockholm, Sweden; ^3^ Department of Cellular Therapy and Allogeneic Stem Cell Transplantation, Karolinska University Hospital and Division of Hematology, Stockholm, Sweden; ^4^ Department of Medicine Huddinge, Karolinska Institutet, Stockholm, Sweden

**Keywords:** cytomegalovirus, WT1 = Wilms tumor 1, immune reconstitution, stem cell transplantation, acute myeloid leukemia (AML)

## Abstract

In allogeneic hematopoietic cell transplantation (allo-HSCT), both virus-specific T cells and leukemia-specific T cells need to be reconstituted to protect patients from virus infections and primary disease relapse. Cytomegalovirus (CMV) infection remains an important cause of morbidity and mortality after allo-HSCT. Emerging data indicate that CMV reactivation is associated with reduced risk of leukemia relapse in patients with acute myeloid leukemia (AML) undergoing allo-HSCT. In a cohort of 24 WT1+ AML patients during the first year following HSCT, CMV specific CD8+ T cells (CMV-CTL) reconstituted much faster than WT1-specific CD8+ T cell (WT1-CTL) after allo-SCT. Moreover, CMV-CTL expressed lower levels of exhaustion markers and were more functional as identified by production of IFN-γ/TNF-α and expression of Eomes/T-bet. Interestingly, our patients with CMV reactivation presented higher frequency of CMV-CTL, lower levels of Eomes+T-bet- and higher levels of Eomes+T-bet+ expression in response to WT1 and CMV pp65 antigen during the first year after transplantation as compared to patients without CMV reactivation. Kinetics of CMV-CTL and WT1-CTL after transplantation might be associated with measurable residual disease and later leukemia relapse. Our results support that CMV reactivation, aside from the CMV-CTL reconstitution, could influence WT1-CTL reconstitution after allo-HSCT, thus potentially contributing to the remission/relapse of AML.

## Introduction

Allogeneic hematopoietic stem cell transplantation (allo-HSCT) is an important curative therapy for patients with high-risk acute myeloid leukemia (AML), but relapse of the disease remains a critical therapeutic challenge with rates of 40-70% (1, 2) and the great majority remain destined to die of resistant disease (3, 4). Abundant evidence supports the presence of the graft-versus-leukemia (GVL) effect after allo-HSCT for AML. It has been shown that antigen-specific T cells targeting Wilms’ Tumor 1 (WT1) have the potential to promote GVL effect without inducing graft-versus-host disease (GVHD) (5–9). WT1 is expressed on a majority of AML cells, and expression of WT1-mRNA in peripheral blood and bone marrow is regarded as a marker of measurable residual disease of AML. Furthermore, WT1-specific T cells (WT1-CTL) can be observed in patients, and these T cells were correlated with antitumor effects after allo-HSCT and after vaccination (10–12). *In vitro* studies and WT1 peptide vaccine trials of patients with leukemias and various types of solid tumors have demonstrated that cytotoxic activity of WT1-specific CD8+ T cells can be induced by these stimuli (13–15). Promising strategy by T cell receptor gene therapy targeting WT1 seemingly could prevent leukemia relapse in AML patients after transplantation (16).

Cytomegalovirus (CMV) infection remains another challenging obstacle in transplant recipients (17). Prophylactic and preemptive antiviral treatment strategies has greatly reduced the morbidity and mortality of CMV infection post allo-HSCT, but the reconstitution of CMV-specific CTL (CMV-CTL) following transplantation is essential to confer long-term protection against CMV infection (18, 19). The interplay between CMV infection and primary disease relapse in patients with hematological malignancies after allo-HSCT has been an area of interest and controversy for several years. Increased evidence and our recent meta-analysis (20) implied that CMV reactivation is associated with a decreased risk of leukemia relapse in AML patients (21–25), although registry data from both the EBMT (26) and the CIBMTR (27) do not support the protective effect of CMV serology or reactivation against relapse.

Possible mechanisms behind decreased AML relapse induced by CMV replication after allo-HSCT is currently unknown. CMV infection could hypothetically educate γδ T cells (28), NK cells (29), and/or tumor-specific T cells. Leukemic blasts could harbor CMV and activated donor-derived T cells could therefore react against these blasts expressing CMV antigens (18). In contrast to other virus-specific T cells, CMV infection has a remarkable dominant influence on the memory T cell compartment. Furthermore, Brodin et al. have elegantly described that CMV profoundly shapes the immune system of healthy individuals and patients after allo-HSCT (30, 31). It suggests that repeated environmental influences such as CMV impose a profound impact on immune homeostasis and dynamic changes in the immune system, particularly on T cells including anti-tumor immunity (32).

Altogether, this indicates that CMV could influence anti-CMV and anti-leukemia T-cell immunity in AML patients. To explore this hypothesis, we compared CMV-specific and WT1-specific T cells in peripheral blood of AML patients after transplantation to determine whether CMV-induced immune response impacts relapse development after allo-HSCT.

## Materials and methods

### Patients

Adult HLA-A*0201-positive patients with AML in first (n=22) or second complete remission (n=2), who had a human leukocyte antigen (HLA)-identical sibling donor or an HLA-A-, HLA-B- or HLA-DRB1- allele-matched (6/6) unrelated donor (MUD) were included in this study. All of the patients were identified as WT1 positive prior to allo-HSCT as described previously (33). WT1 was not measured after allo-HSCT or investigated in the donors. Peripheral blood mononuclear cells (PBMCs) were obtained from AML patients after IRB approval (Stockholm Ethical Committee South 2010/760-31/1) and informed consent. PBMCs were isolated by the Ficoll-Hypaque density gradient at 1, 2, 3, 6, and 12 months after transplantation and preserved in liquid nitrogen in fetal bovine serum (Gibco, Invitrogen) containing 10% dimethysulfoxide until use. Seven healthy controls´ samples were obtained from the LifeGene study (2009/1183-31) after they signed informed consent. Samples availability per donor during the follow-up is shown in **Supplementary Table 1**.

Myeloablative conditioning (MAC) was given to 7 patients and reduced-intensity conditioning (RIC) was given to 17 patients. Immunosuppressive treatment consisted mainly of cyclosporine A (CsA) in combination with a short course of methotrexate (n=20) or tacrolimus and sirolimus (n=4). The nineteen patients with an unrelated donor received antithymocyte globulin (ATG, Thymoglobulin; Genzyme, MA) for 2 to 4 days during conditioning. Standard prophylaxis against infections included fluconazole, ciprofloxacin, acyclovir, and trimethoprim/sulfamethoxazole. Weekly surveillance for CMV DNAemia and preemptive therapy was given as described previously (34, 35).

Disease indication in combination with disease stage at the time of HSCT were used for the disease risk index classification (36), to group patients into the low-, intermediate-, high-, or very high risk patient cohorts. CMV infection was defined as positive CMV PCR, and CMV disease was defined according to Ljungman et al. (37). Acute and chronic GVHD was diagnosed using established criteria and grading (38, 39). Hematological relapse was defined as bone marrow (BM) blasts ≥ 5%, appearance of blasts in peripheral blood, and/or detection of extramedullary disease (40–42).

### Donor-recipient chimerism analysis and MRD analysis after HSCT

A quantitative PCR-based analysis of short tandem repeats was performed at 1, 3, 6 and 12 months after allo-HSCT. PCR was routinely used to evaluate the degree of donor and recipient chimerism in CD19+, CD3+ and CD33+ cells enriched from blood, and CD34+, CD33+, CD19+, and CD3+ cells in bone marrow using magnetic beads (Dynal, Oslo, Norway) as previously described (43, 44). Full donor chimerism was defined as >95% donor-derived cells in all lineages. Mixed chimerism was defined when >5% but <95% donor-derived cells were present in at least 1 cell lineage.

Measurable residual disease (MRD) assessments with flow cytometry were performed regularly in bone marrow and scheduled at the same intervals as the chimerism analyses when possible. Leukemia-associated immunophenotypes (LAIP) were identified in bone marrow specimens. Positive flow cytometry was defined as ≥ 0.05% in AML patients after transplantation if the LAIP was distinctive.

### Determination and characterization of CMV-specific and WT1-specific CD8+ T cells by MHC-Dextramer analyses

CMV-specific T cells (CMV-CTL) and WT1-specific T cells (WT1-CTL) frequencies were quantified and phenotyped in patients by staining with PE-A^*^0201 CMV_NLVPMVATV_ Dextramer, PE-A^*^0201 WT1_RMFPNAPYL_ Dextramer and APC-A^*^0201 WT1_SLGEQQYSV_ Dextramer (Immudex, Copenhagen, Denmark) (**Supplementary Figure 1**). Briefly, PBMCs were stained with Dextramers for 30 min at room temperature. Anti-CD3-Brilliant Violet 570, anti-CD4-Brilliant Violet 510, anti-CCR7-Brilliant Violet 421, anti-CD45RA–FITC, anti-PD-1-PE Cy7 (from Biolegend, San Diego, CA, USA), anti-CTLA-4–PE Cy5, anti-CD8-AF700 (from BD Biosciences, San Jose, California, USA) and anti-TIM-3–PerCP eFlour 710 (from eBioscience, USA) were added for the final 20 minutes of incubation. Surface expression of CD45RA and CCR7 was used to characterize naïve (T_Naive_, CD45RA+CCR7+), central memory (T_CM_, CD45RA-CCR7+), effector memory (T_EM_, CD45RA-CCR7-), and terminally differentiated (T_EMRA_, CD45RA+CCR7-) phenotypes (**Supplementary Figure 2**). Appropriate isotype controls or fluorescence minus one control for each fluorochrome were used to assess for nonspecific staining and determine gating strategy, respectively. FACS Aria flow cytometer (BD Biosciences, San Jose, California, USA) and FlowJo software were used for acquisition and data analysis.

### Intracellular staining of T cells and CD107a induction assay

The proportion and phenotype of CD8+ or CD4+ T cells producing IFN-γ and TNF-α in response to stimulation with the CMV pp65 protein or WT1 protein (15 mers, with 11 aa overlap, swiss prot: P06725 and P19544; Peptides&Elephants) were measured by FACS analysis as previously described (45, 46). One million PBMCs were incubated with or without peptides in the presence of Brefeldin-A (10 mg/ml; Sigma-Aldrich) for 5h. Stimulation was stopped by keeping the cells at 4°C overnight. Subsequently, T cells were stained with anti-CD3-ECD (Beckman Coulter, CA, USA), anti-CD4-PerCP-Cy5.5, anti-CD8a-AF700, anti-TNF-α-APC, anti-IFN-γ-PE-Cy7 (from BD Biosciences, San Jose, California, USA), anti-Eomes FITC, and anti-T-bet PE (from eBioscience, USA). FACS Aria flow cytometer was used for acquisition, and data analysis was performed using FlowJo software.

Upon antigen stimulation, cytolytic activity of T cells was measured by degranulation assay. Cells pre-stained with CD107a antibody (APC AF700, BD Biosciences, San Jose, CA, USA) were cultured with different antigens (PMA as the positive control) for 5 hours in the presence of monensin (BD Biosciences, San Jose, CA. USA). Cells were then stained with anti-CD3-Brilliant Violet 570, anti-CD4-Brilliant Violet 510 (from Biolegend, San Diego, CA, USA) and anti-CD8-APC Cy7 (BD Biosciences, San Jose, California, USA). Unstimulated cells for each sample were used for spontaneous degranulation (baseline). Cells were analyzed by flow cytometry as described above.

### Generation, isolation, and maintaining of CMV-specific T cells

CMV-specific T cell line (HD-2) and T cell clones (A1-8 and A1-9) specific for CMV pp65 protein were generated from a single HLA-A*0201+ healthy control. CMV-specific T cells were stained with HLA-PE-conjugated A*0201 CMV_NLVPMVATV_ Dextramer (Immunex, Copenhagen, Denmark) for 30 min at room temperature. HLA-A*0201-CMV-CTL were then sorted using a FACS Aria flow cytometer.

Sorted CMV-CTL from HD-2 cell line were then clonally selected using limiting dilution in T cell medium containing Serum-free medium (Cell-Genix, Freiburg, Germany), with 10% human AB serum (Innovative Research, MI) supplemented with IL-2 (1000 IU/ml), IL-15 (10ng/ml), IL-21 (10ng/ml), (Prospec, Ness-Ziona, Israel), penicillin (100IU/ml), streptomycin (100μg/ml) (Life Technologies, Carlsbad, CA), and amphotericin B (2.5mg/L) (Sigma-Aldrich, St Louis, MO) and seeded with anti-CD3 (OKT3) (Biolegend, CA, USA) and addition of γ-irradiated allogeneic feeder cells at a 5:1 E:T ratio. After 14 days, the clonal selection was repeated. Expanded T cell clones were transferred into larger vessels when required where they were subsequently expanded in T cell culture medium and maintained by biweekly stimulation with OKT3 and irradiated allogenic feeder cells.

### Mapping of immunogenic epitopes of WT1 recognized by CMV-specific T cells

Aliquots of CMV-specific T cells (A1-8 and A1-9 cell lines) were stimulated with individual peptides of WT1 (5μg/ml) for 72h. Survivin peptides (15 mers, with 11 aa overlap, swiss prot: O15392; Peptides&Elephants) were used as a control. After 72 h stimulation, supernatants were collected and concentration of IFN-γ was evaluated by ELISA according to the manufacturer’s instructions (Mabtech, Nacka Strand, Sweden). The mapping grid was used to identify specific WT1 15-mers eliciting T-cell response. Identified specific WT1 peptides were then used for intracellular cytokines staining as described above to confirm their specific immunogenicity.

### Statistical analysis

Data were analyzed by X^2^ test for categorical data and Mann-Whitney U-test for continuous data. Two-way ANOVA was used to analyze differences between two groups over time after HSCT. We used Wilcoxon matched-pairs signed rank test or Friedman test to detect differences between two matched timepoints or repeated matched time points. Tukey’s multiple comparisons test was used for pairwise comparisons if significant. Results are shown as the mean ± standard deviation (SD) or as the mean ± SEM when repeated sampling was considered. A value of p from 2-sided tests less than 0.05 was considered statistically significant. STATA software (STATA, College Station, TX) and GraphPad Prism 6 software (GraphPad Software, Inc., La Jolla, CA, USA) were used for data analysis.

## Results

### Patients

Twenty-four patients with AML were longitudinally monitored after allo-HSCT, and their characteristics are shown in **Table 1**. Of the 24 patients, 10 were male and 14 were female. The median age at transplantation was 56.5 years (range, 21-72). Four patients underwent transplantation from an HLA-matched sibling, and 20 from a matched unrelated donor. Sixteen of 24 patients were alive at the time points shown. Seven patients developed hematological leukemia relapse of whom 6 died during the follow-up. Five of 7 relapses occurred during the first year after transplantation, and the median time to relapse was 278 days (range 88-760). Donor lymphocytes infusion (DLI) were administered to 5 patients, and the median time from transplantation to first DLI was 378 days (range 78-994). Sixteen of 24 patients had CMV reactivation at a median of 25.5 days after HSCT (range 16-1032), but none developed CMV disease. Five of 16 patients with CMV reactivation relapsed, while 2 of 8 patients without reactivation developed leukemia relapse (p = 1.00).

**Table 1:** Patients characteristics.

ID	Diagnosis	Age, year	Sex, P/D	Donor	CMV serostatus, P/D	DRI	CMV reactivation, day	Survival	DLI days post-HSCT, dose, reason	Relapse, day	aGVHD, day, grade (site)	cGVHD, day, grade
1	AML	29	M/M	MUD	(+/+)	IM	Y, 18	Dead(leukemia)	78, 3, preemp	Y, 308	N	N
2	AML	54	M/M	MRD	(+/-)	IM	Y, 46	Dead(cardiac arrest)	N	N	17, II (skin, liver)	211, severe
3	AML	36	F/F	MRD	(+/-)	IM	N	Alive	N	N	29, II (gut)	N
4	AML	51	F/M	MUD	(+/+)	IM	Y, 18	Alive	571, 6, preemp	N	N	N
5	AML	60	F/F	MUD	(+/+)	Low	Y, 25	Dead(adenocarcinoma)	377, 4, thera	Y, 276	N	572, mild
6	AML	45	F/F	MUD	(-/-)	IM	N	Alive	N	N	N	271, mild
7	AML	53	F/M	MUD	(+/+)	High	Y, 17	Alive	N	N	62, II (gut)	N
8	AML	60	M/M	MUD	(-/-)	High	N	Dead(leukemia)	N	Y, 88	73, I (skin)	N
9	AML	38	F/F	MUD	(+/+)	IM	Y, 24	Alive	N	N	21, II (skin)	N
10	sAML	58	M/M	MUD	(+/+)	IM	Y, 31	Dead(leukemia)	N	Y, 462	11, II (gut)	277, mild
11	AML	60	F/F	MUD	(+/+)	IM	Y, 18	Alive	N	N	59, I (skin)	241, mild
12	AML	57	M/M	MRD	(+/+)	IM	Y, 109	Alive	N	N	81, III (skin, liver, gut)	363, severe
13	AML	68	F/F	MUD	(+/+)	IM	Y, 34	Alive	N	N	113, I (skin)	N
14	AML	40	M/M	MUD	(+/+)	IM	Y, 1032	Dead(leukemia)	994, 1, therap	Y, 760	N	106, mild,
15	AML	49	F/F	MUD	(-/-)	IM	N	Alive	N	N	30, I (skin)	304, mild,
16	AML	26	M/F	MUD	(+/-)	High	Y, 16	Alive	N	N	20, II (gut)	N
17	sAML	62	M/M	MUD	(+/+)	IM	N	Alive	N	N	56, I (skin, liver)	272, mild
18	AML	64	F/M	MUD	(+/+)	IM	Y, 32	Alive	N	N	25, I (skin)	125, mild
19	AML	56	M/M	MUD	(-/-)	IM	N	Alive	N	N	N	N
20	AML	72	F/M	MRD	(+/+)	IM	N	Alive	N	N	N	mild, 95
21	AML	63	M/M	MUD	(+/-)	IM	Y, 53	Alive	N	N	5, II (gut)	moderate, 96
22	AML	69	F/F	MUD	(+/+)	IM	Y, 25	Alive	N	N	23, II (skin, gut)	N
23	AML	21	F/M	MUD	(+/-)	IM	N	Dead(leukemia)	378, 2, therap	Y, 278	11, I (skin, gut)	N
24	AML	61	F/M	MRD	(+/-)	High	Y, 26	Dead(leukemia)	N	Y, 208	47, I (skin)	N

AML, acute myeloid leukemia; sAML, secondary AML; M, male; F, female; MUD, matched unrelated donor; MRD, matched related donor; P, patients; D, donors; DRI, disease risk index; IM, intermediate; CMV, cytomegalovirus; DLI, donor lymphocytes infusion; aGVHD, acute graft-versus-host disease; cGVHD, chronic graft-versus-host disease; Y, yes; N, no; preemp, preemptive therapy; therap, therapeutic.

### CMV-specific and WT1-specific T cells reconstitution in AML patients after allo-HSCT

**Figure 1:**
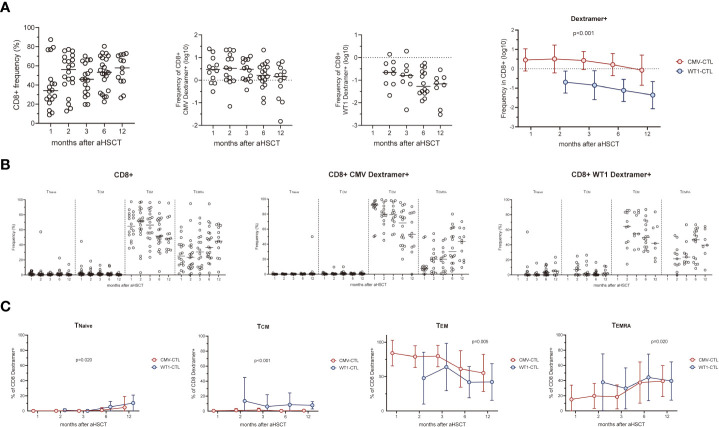
CMV-specific and WT1-specific CD8+ T cells reconstitution quantified by MHC-Dextramer analyses in HLA-A*0201-expressing AML patients after HSCT. Mean values ± SD at each time point are represented. **(A)** CD8+, CMV- and WT1-specific CD8+ T cells reconstitution overtime after HSCT. **(B)** Longitudinal phenotypic characterization of CD8+ T cells, CMV- and WT1-specific T cells based on the expression of CD45RA and CCR7. **(C)** Comparison of memory compartment within CMV- and WT1-specific T cells. Two-way ANOVA was used to detect differences between two groups over time. T_Naive_, CD45RA+CCR7+, T_CM_, CD45RA-CCR7+, T_EM_, CD45RA-CCR7- and T_EMRA_, CD45RA+CCR7-.

In our cohort, while not significant, there was a gradual increase frequency of CD8+ T cells from 39.77 ± 5.78% (mean ± SEM) at month 1 to 55.37 ± 4.49% at month 12 after allo-HSCT (**Figure 1A**). CMV-CTL were detectable in 18 of 20 patients with D+ and/or R+ after HSCT, with frequencies between 0.015% and 24.20% of the CD8+ T cell subpopulation. WT1-CTL were detectable in 19 of 24 patients after HSCT, with frequencies between 0.01% and 1.91% of the CD8+ T cell subpopulation. No WT1-CTL was found on month 1 post-HSCT. CMV-CTL reconstituted to a higher extend than WT1-CTL (p<0.001, **Figure 1A**). Overtime, CD8+ T cells exhibited a predominant but decreasing T_EM_ phenotype and increasing T_EMRA_ phenotype (**Figure 1B**). Although both WT1/HLA-A*0201+ CD8+ T cells and CMV/HLA-A*0201+ CD8+ T cells resided predominantly in T_EM_ and T_EMRA_, a larger population of T_EM_ was observed in the CMV-CTL during the first year after allo-HSCT as compared to WT1-CTL (p=0.005). Reversely, higher proportion of T_Naive_ (p=0.020), T_CM_ (p<0.001) and T_EMRA_ (p=0.020) phenotypes were observed in WT1-specific T cells as compared to CMV-CTL (**Figure 1C**). This suggested an ongoing *de novo* generated immune response to WT1, with a shift toward a more T_Naive_ and T_CM_ phenotype later following allo-HSCT.

**Figure 2:**
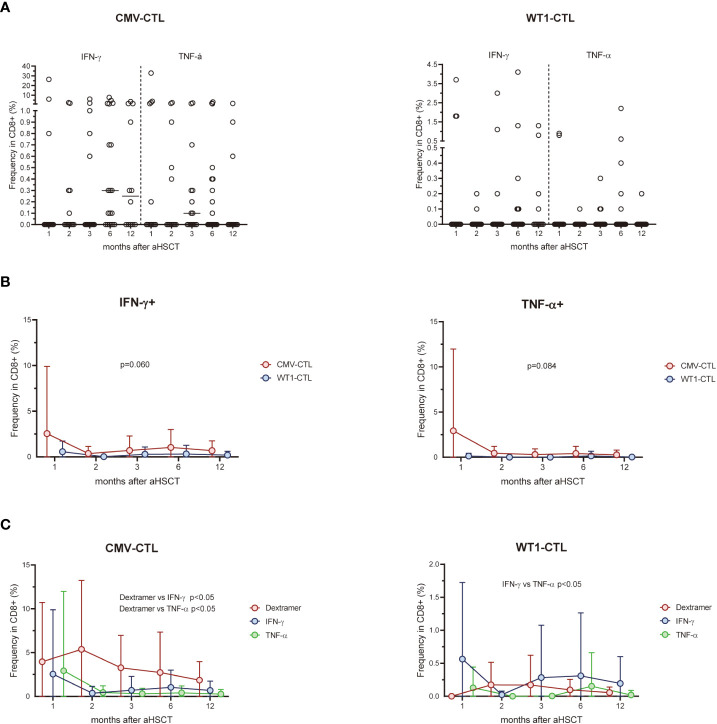
**(A)** Percentage of CD8-positive WT1-CTL or CMV-CTL were quantified by intracellular IFN-γ and TNF-α production in response to WT1 or CMV peptides in AML patients at 1, 2, 3, 6, and 12 months after transplantation. **(B)** There was a trend for more IFN-γ or TNF-α producing CMV-CTL than WT1-CTL during the first year post-SCT. **(C)** The frequency of Dextramer+ CMV-CTL in transplant recipients were higher than that of IFN-γ+ or TNF-α+ CMV-CTL from 1 month after allo-HSCT, while the frequency of TNF-α+ WT1-CTL were lower than that of IFN-γ+ WT1-CTL during the first year after transplantation. Graphs present mean values ± SD at each time point. Two-way ANOVA was used to detect differences between two groups over time.

We next analyzed the T cell response to CMV pp65 protein or WT1 protein by intracellular IFN-γ/TNF-α production at 1, 2, 3, 6, and 12 months post-HSCT (**Figure 2A**). Responses were detected in 18 (IFN-γ) and 13 (TNF-α) of 20 patients with D+ and/or R+ stimulated by CMV pp65, and 17 (IFN-γ) and 8 (TNF-α) of 24 patients stimulated with WT1. In 12 out of 20 patients, CD8+ T cells produced both IFN-γ and TNF-α in response to CMV pp65, whereas this was found in only 7 out of 24 when using WT1 as a stimulus. There was a trend for more IFN-γ or TNF-α producing CMV-CTL than WT1-CTL during the first year post-SCT (**Figure 2B**). After excluding four D-/R- pairs who did not respond to CMV, higher frequency of Dextramer+ CMV-CTL than IFN-γ or TNF-α producing cells was found in the 20 responding patients (p<0.05). Different pattern of WT1-CTL was found with an overall higher frequency of IFN-γ producing cells as compared to TNF-α producing WT1-CTL (p<0.05, **Figure 2C**).

### WT1-specific CD8+ T cells have an increased expression of PD-1, CTLA-4, and TIM-3 compared to CMV-specific CD8+ T cells after transplantation

**Figure 3:**
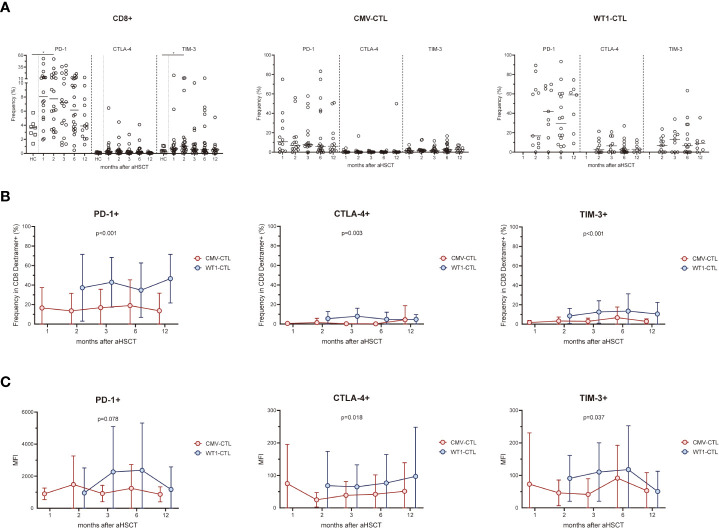
WT1-specific CD8+ T cells have an increased expression of PD-1, CTLA-4, and TIM-3 compared to CMV-specific CD8+ T cells after transplantation. **(A)** Expression of exhaustion molecules PD-1, CTLA-4 and TIM-3 was assessed on CMV-specific and WT1-specific CD8+ T cells after transplantation. Graphs present mean values ± SD at each time point. PD-1 and TIM-3 on CD8+ T cells in the transplant recipients was higher at 2 months after HSCT than that from healthy donors, and no difference was found in term of CTLA-4 expression. **(B)** Frequencies of PD-1+, CTLA-4+, and TIM-3+ cells among WT1-specific CD8+ T cells were significantly higher than those of CMV-specific CD8+ T cells after transplantation. **(C)** Mean fluorescence intensity (MFI) of PD-1, CTLA-4, and TIM-3 of CMV-CTL was overall higher than those of WT1-CTL after transplantation. The Kruskal-Wallis test followed by Dunn’s post-test was used to compare patients with controls. Comparisons between different groups were made using the Mann–Whitney test and two-way ANOVA was used to detect differences between two groups over time. HC, healthy controls. *p < 0.05.

We assessed the expression of the activation/inhibitory molecules PD-1, CTLA-4, and TIM-3 on the cell surface of CD8+ T cells and antigen-specific substets of the AML patients after allo-HSCT and healthy controls. Higher surface expressions of PD-1 and TIM-3 on CD8+ T cells in the early months post-HSCT were observed in patients, especially at month 2 (p=0.031 and p=0.046 respectively), as compared to the frequency found in the healthy controls. Overtime post-HSCT, AML patients did not show significant differences in the surface expression of PD-1, CTLA-4, and TIM-3 within CMV-CTL or WT1-CTL (**Figure 3A**). Interstingly, frequencies of PD-1, CTLA-4, and TIM-3 cells in WT1-specific CD8+ T cells were remarkably higher than those of CMV-specific CD8+ T cells after transplantation (p<0.001, p=0.003, p<0.001) (**Figure 3B**). The mean fluorescence intensity (MFI) of PD-1, CTLA-4, and TIM-3 was overally higher than those of CMV-specific CD8+ T cells overtime post-SCT (p=0.078, p=0.018, p=0.037) (**Figure 3C**).

### Expression of the transcription factors Eomes and T-bet in CMV-specific and WT1-specific T cells correlates with differential cytokine expressing patterns

**Figure 4:**
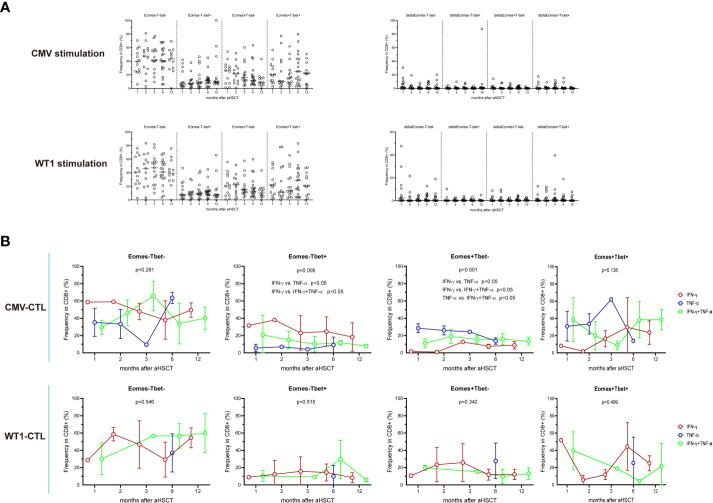
Expression of the T-box transcription factors Eomes and T-bet in CMV-specific and WT1-spcific CD8+ T cells. PBMCs from transplant recipients were stimulated with CMV pp65 or WT1 protein overnight, stained with anti-CD8 and assessed for intracellular expression of IFN-γ, TNF-α, Eomes, and T-bet. IFN-γ and/or TNF-α producing cells are shown as an overlay of total CD8 T cells. Graphs present mean values ± SD at each time point. **(A)** indicates longitudinal pattern of Eomes/T-bet expression and delta of Eomes/T-bet expression (Specific Eomes/Tbet expression during antigen stimulation -Eomes/Tbet expression in unstimulated cells) by CMV-specific or WT1-specific CD8 T cells. **(B)** shows frequency of IFN-γ+ or TNF-α+ or IFN-γ+TNF-α+CMV-specific and WT1-specific T cells expressing different profiles of Eomes and T-bet (Eomes-T-bet-, Eomes-T-bet+, Eomes+T-bet-, or a Eomes+T-bet+). Eomes and T-bet profiles were compared among IFN-γ+ or TNF-α+ or IFN-γ+TNF-α+. IFN-γ+CD8+ T cells showed highest proportion of Eomes-T-bet+ and lowest proportion of Eomes+T-bet- (p<0.05), while TNF-α+CD8+ T cells instead indicated lowest proportion of Eomes-T-bet+ and highest proportion of Eomes+T-bet- (p<0.05). Graphs present mean values ± SD at each time point. Comparisons were made using the Friedman test followed by Dunn’s post-test over time after HSCT.

We then investigate the possibility that selective production of IFN-γ and/or TNF-α by CTL may be intrinsically linked to the expression of Eomes and T-bet: AML patients’ PBMCs from different timepoints post-HSCT (1M, n=13; 2M, n=13; 3M, n=15; 6M, n=19; 12M, n=12) were stimulated with CMV or WT1 peptides and then measured the expression of these transcription factors and the production of IFN-γ and/or TNF-α. Four patterns of expression for Eomes and T-bet: Eomes-T-bet-, Eomes+ T-bet-, Eomes- T-bet+, and Eomes+ T-bet+ were observed, but about 41% of the CD8+ T cells regardless of antigen stimulation did not express Eomes and T-bet (mean ± SEM: 41.6 ± 2.481% and 41.7 ± 2.293% respectively, **Figure 4A** and **Supplementary Figure 3**). After stimulating with CMV antigens, IFN-γ+CD8+ T cells showed highest proportion of Eomes-T-bet+ and lowest proportion of Eomes+T-bet- (p<0.05), while TNF-α+CD8+ T cells instead indicated lowest proportion of Eomes-T-bet+ and highest proportion of Eomes+T-bet- (p<0.05) and IFN-γ+TNF-α+ CD8+ T cells were in the intermediate level (**Figure 4B**). By contrast, no difference was found in CD8+ T cells stimulated with WT1 antigens producing IFN-γ and/or TNF-α in terms of Eomes/T-bet expression. Thus, distinct profiles of Eomes/T-bet expression was found for antigen-specific T cells, T-cells that displayed Eomes-T-bet+ phenotype or T-cells that expressed Eomes+T-bet- phenotype in regard to production of IFN-γ/TNF-α, with increased levels of Eomes and T-bet strongly correlated with differentiation from central memory to effector memory and effector subpopulations (47).

### CMV infection affects CMV- and WT1-specific T cells

**Figure 5:**
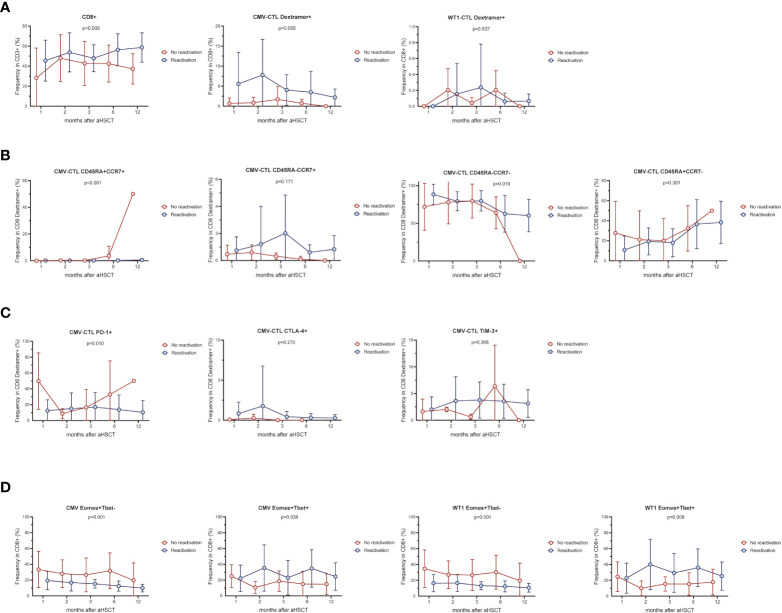
CMV infection affects CMV-specific and WT1-specific T cells. The frequency of CMV-CTL or WT1-CTL identified by Dextramer **(A)** and their memory compartment **(B)** in patients with CMV reactivation was compared to that of patients without CMV reactivation during the first year after allo-HSCT. Exhaustion marker PD-1, CTLA-4, and TIM-3 **(C)** and Eomes/T-bet expression **(D)** was also compared in terms of CMV reactivation or not. Graphs present mean values ± SD at each time point. Two-way ANOVA was used to detect differences between two groups over time.

AML patients with CMV reactivation (n=16) presented higher frequency of CD8+ T cells and CMV-CTL (Dextramer positive) (p=0.009, p=0.005) during the first year post-HSCT compared to patients without reactivation (n=8) (**Figure 5A**). No difference between the 2 groups were found in the frequency of Dextramer+WT1-CTL and IFN-γ/TNF producing CD8+ T-cells stimulated by CMV/WT1 antigens during the first year after allo-HSCT (**Figure 5A** and **Supplementary Figure 4A**). Furthermore, while the memory phentype of CD8+ T cells and WT1-CTL were not impacted by the CMV reactivation (**Supplementary Figure 4B**), CMV-CTL of patients with CMV reactivation presented a constantly lower frequency of naïve phenotype (p<0.001) and a higher frequency of an effector memory phenotype (p=0.019) than patients without CMV reactivation (**Figure 5B**). Interestingly, lower and stable PD-1 expression on CMV-CTL (P=0.01) was found in patients with CMV reactivation (**Figure 5C**), but CMV reactivation did not impact the expression of activation and exhaustion markers on CD8+ T cells and WT1-CTL (**Supplementary Figure 4C**).

Overtime post-HSCT, lower proportion of Eomes+T-bet- CD8+ T cells and higher proportion of Eomes+T-bet+CD8+ T cells were observed in response to CMV or WT1 stimulation (p<0.001 and p=0.028, p<0.001 and p=0.009, respectively) in patients developing CMV reactivation after allo-SCT, although only one sample presented a detectable WT1-specific immune response at month 1 in patients without CMV reactivation (**Figure 5D**).

### Kinectics of CD8+ T-cell responses to CMV and WT1 and disease activity

**Figure 6:**
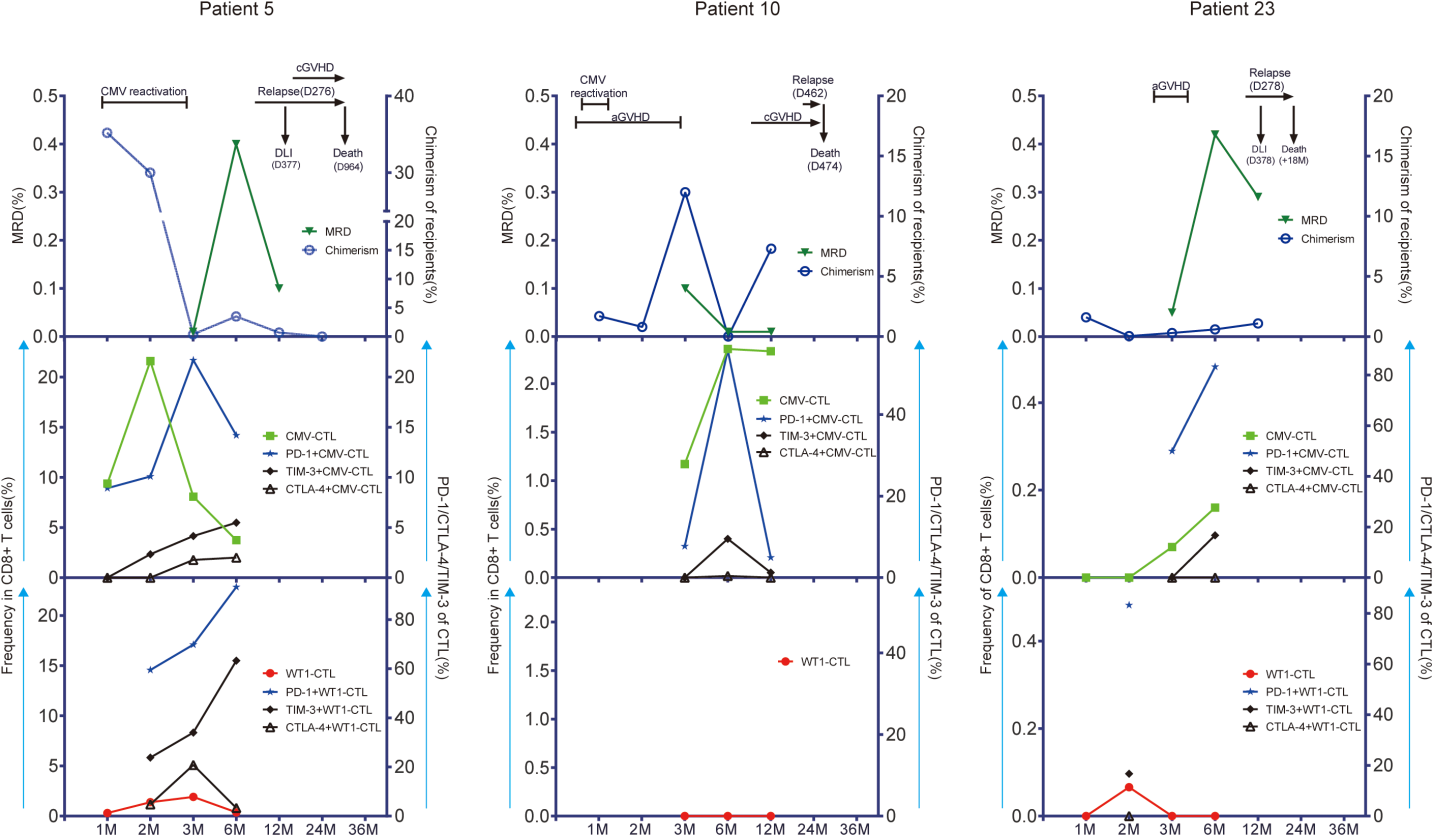
CMV-CTL and WT1-CTL responses in peripheral blood in relation to disease activity as measured by measurable residual disease (MRD) and recipients chimerism. Chimerism status was determined within CD33+ separated cells in bone marrow. Results in 3 individual patients with detectable WT1 before HSCT are shown (Patient 5; Patient 10; Patient 23). The number of months (M) after transplantation is shown on the x-axis. CMV-CTL and WT1-CTL are expressed as percentage of CD8+ T cells in peripheral blood (middle/low left, y-axis: green square/red solid circle). PD-1, TIM-3, and CTLA-4 are expressed as percentage of CMV-CTL/WT1-CTL (middle/low right, y-axis: blue solid star/solid black rhombus/black triangle/). Disease activity is expressed as MRD identified in bone marrow specimens by flow cytometry (up left, y-axis: triangle in dark green) and recipients chimerism (up right, y-axis: blue circle). Times of donor lymphocytes infusion (DLI), CMV reactivation, graft-versus-host disease (GVHD), and leukemia relapse are depicted on each graph. Patient 5 was a 60-year-old woman with AML in second remission (CR2). WT1-CTL were increasingly detected in the peripheral blood for the first 90 days after allo-HSCT. This was associated with decreasing recipient chimerism and negative MRD. She developed CMV reactivation during the first month and had good response to Ganciclovir. However, WT1-CTL decreased up until 6 months, which coincided with rising recipient chimerism, and the peak of MRD. This patient relapsed with 70% blasts in bone marrow at day 276. She received chemotherapy and 4 doses of DLI, went into morphologic remission, but died due to a secondary malignancy 2.5 years after allo-HSCT. Although the frequency of CMV-CTL decreased after month 2, the expression of PD-1, TIM-3,or CTLA-4 on CMV-CTL/WT1-CTL were upregulated persistently before leukemia relapse. Patient 10 was a 58-year-old man with secondary AML in CR1. He developed grade 2 acute GVHD of the gut at day 11, which was treated successfully with a short course of steroids during the first month after transplantation. He experienced a CMV reactivation which was treated with Ganciclovir with good response. During this time, WT1-CTL was not detectable in peripheral blood, while more than 1% of the CD8+ T cells were CMV-CTL. Recipient chimerism rose after 6 months, and although he developed chronic GVHD in the mouth, eye, and skin at 8 months, he had leukemia relapse at 15 months with blasts in bone marrow, and died at day 474 after allo-HSCT. Patient 23 was a 21-year-old woman with AML in CR1. She developed grade 1 aGVHD in skin without CMV reactivation after allo-HSCT and had good response to steroids. WT1-CTL appeared transiently at month 2 and then lost, while CMV-CTL increased steadily with high expression of PD-1 and TIM-3 on CMV-CTL, and this patient had leukemia relapse at day 278. She received chemotherapy and DLI with no effect and died at 18 months after transplantation.

Only higher expression of CTLA-4 on CD8+ T cells was observed in patients with relapse (n=7) during the first year after allo-SCT (p=0.021) as compared to patients without leukemia relapse (n=17). No other difference in CMV-specific, WT1-specific T cells and associated studied phenotypes was observed between the 2 patient groups defined by relaspe occurence (**Supplementary Figures 5, 6**). As low number of patients relaped in this cohort (n=7), we then look especially into three individual patients with leukemia relapse who had samples of more than 3 time points after transplantation available for this analysis (Patient 5, 10, and 23). We monitored the kinetics of CMV-specific and WT1-specific CD8+ T-cell responses and associated activation/exhaustion markers together with chimerism and disease regression for each time point where samples were available. Patient 5 and 10 developed CMV reactivation, but Patient 23 did not. As shown in **Figure 6** decreased or lost WT1-CTL responses (red symbols and line) in these three patients was followed by a reemergence of leukemia as measured by MRD (green symbols and line) and chimerism (blue symbols and line). Interestingly, in these 3 patients, the pattern of CMV-CTL apparently changed inversely to that of WT1-CTL, and high expression of TIM-3 and PD-1 on CMV-CTL or WT1-CTL seemed to correlate with positive MRD and later leukemia relapse in two of them.

A different pattern in antigen-specific immunity was observed in 15 patients without leukemia relapse (**Supplementary Figures 7–11**): Higher levels of WT1-specific immune responses (>= 0.5% of CD8+ T cells) were found in three patients (Patient 3, 6, and 7) in complete remission, while WT1-specific T cells remained undetected in 4 cases (Patient 9, 11, 12, and 16) or at low levels in 8 cases (Patient 2, 4, 13, 17, 18, 20, 21, and 22) with variable expression of PD-1, CTLA-4 and TIM-3. Notablely, CMV-specific T cells were decreased and less exhausted in terms of PD-1, CTLA-4 and TIM-3 expression over time in most of these 15 patients. Patient 15 and 19 were not represented as one did not present antigen-specific immune responses by flow cytometry and the second (D-/R-) had no detectable CMV-specific immunity.

### Potential cross-reactivity between CMV-specific T cells and WT1

**Figure 7:**
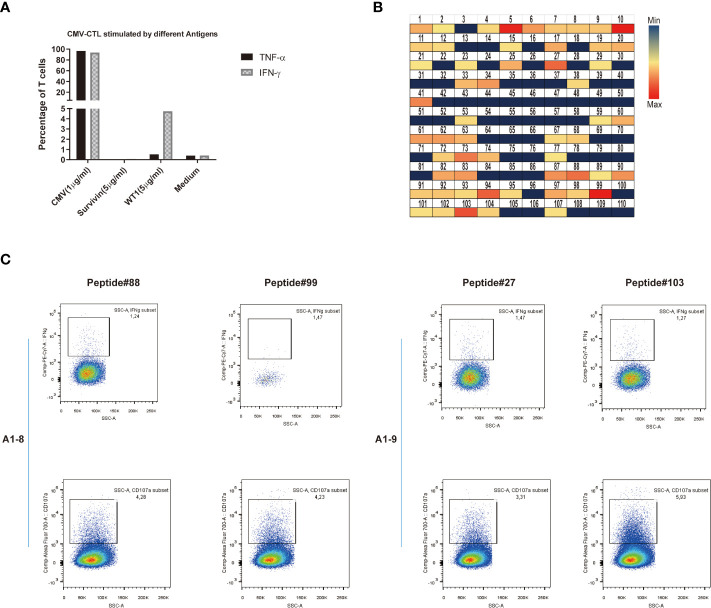
Potential cross-reactivity between CMV-specific T cells and WT1. **(A)** IFN-γ or TNF-α production of CMV-CTL stimulated by different antigens (CMV, Survivin, or WT1 peptides). **(B)** Heat map representation of amino acid diversity across the full-length WT1 genome, illustrating the cross-reactivity of CMV-CTL against WT1. Each square represents a single peptide, colored to reflect the levels of supernatant IFN-γ released by CMV-CTL (median, min-max: 0.217, 0-137.92 pg/ml). **(C)** Data presented show that IFN-γ production and CD107a mobilization of CMV-specific T cell clones (A1-8, A1-9) stimulated by individual WT1 peptides (WT1 peptides 27, 88, 89, and 103). It was assessed by stimulation and intracellular staining of T cells.

Last, we looked at the possible cross-reactivity between the in-house established CMV-specific T cell line (HD-2) and T-cell clone (HD-2-A1-8, 9) with WT1 or survivin, a different unrelated cancer associated antigen. We have co-stained these T cells with CMV dextramer and WT1 dextramer with no WT1 dextramer+ cells found. After incubation of CMV-specific T cells (T-cell clone: HD-2-A1-8, HD-2-A1-9) with different antigens, the specific response was evaluated by intracellular staining (ICS) assay. Only against WT1 at the concentration of 5 μg/ml, up to 5% of CMV-specific T cell clone showed some reactivity by producing IFN-γ (**Figure 7A**). CMV-specific T cells (T-cell clone: HD-2-A1-8, 9) were next co-incubated with 110 individual peptides of WT1 peptides for mapping (**Supplementary Table 2**). Following incubation, strong (median, min-max: 0.217, 0-137.92 pg/ml) IFN-γ production was observed against 11 WT1 peptides (WT1 peptides 5, 10, 27, 41, 73, 87, 88, 90, 94, 99, and 103) which were selected for specific ICS assay (**Figure 7B**). WT1 peptides 88 (GEKPYQCDFKDCERR) and 99 (SRSDHLKTHTRTHTG) induced IFN-γ production and degranulation in clone A1-8 while WT1 petides 27 (GACRYGPFGPPPPSQ) and 103 (TSEKPFSCRWPSCQK) induced IFN-γ production and degranulation in clone A1-9 (**Figure 7C**). Altogether, this suggest that some specific CMV T-cell clones may be more responsible for the possible cross-reactivity with WT1.

## Discussion

Primary disease relapse and CMV infection remain important causes of morbidity and mortality after allo-HSCT. Hence, rapide reconstitution of virus-specific and leukemia-specific T cells after allo-HSCT is crucial to protect patients from virus infections and leukemia relapse. Efficient immune response to pathogens is driven by cytotoxic T cells with high-affinity TCRs. It is unclear whether the reconstitution of CMV-specifc T cells and tumor-antigen specific T cells, two principally different kinds of cell types, is synchronous in quantity and in quality. Our data indicate that CMV- specific T cells reconstituted much faster than WT1-specific T cell after allo-HSCT with the reconstitution visible as early as 30 day post-HSCT. Moreover, CMV-specific T cells expressed lower levels of exhaustion markers and responded more vigourosly to stimuli with intracellular cytokine production. We demonstrated that CMV- specific T cell clones were able to repsond to a leukemia antigen stimulus, WT1, but whether or not CMV infection could affect the leukemia-specific T cells, and whether CMV-specific T cells are able to cross-react against leukemia cells remain to be explored.

CMV regularly contributes to the homeostasis of circulating T cells including global and antigen‐specific immune signatures, both in immunosuppressed individuals and in the immunocompetent host (32). We found that CMV infection was associated with a downregulation of the expression of exhaustion markers on CMV-specific T cells such as PD-1. Accordingly, more functional CMV-specific T cells, characterized by specific cytokines production and transcription factors expression were found in patients with CMV reactivation. Smaller subset of Eomes+T-bet- CD8+ T cells and bigger subset of Eomes+T-bet+ CD8+ T cells with or without WT1/CMV pp65 antigen stimulation were observed in patients with CMV reactivation druing the first year post-HSCT compared to patients without reactivation. CMV in young humans and young mice may enhance immune responses to some antigens including influenza virus;This potential protection may be due to an increased pro-inflammatory responses induced by CMV-specific immune response (48, 49). Primary human CMV systemically induces a Type 1 cytokine signature including interleukin-18, interferon-inducible protein-10, and IFN-γ, which was maintained during latency (50). Similarly to TCR signals, these inflammatory signals, maintenained during latency are also sufficient to regulate the expression of T-bet and Eomes, master regulators of T cell function, and directly affect PD-1 expression in exhausted T cells (51–53). These results showed that CMV may improve the polyfunctionality and consequently the quality of global CD8+ T cell immune response, CMV-specific immunity and newly-generated immune response against leukemia like WT1 in the context of allo-HSCT, although we found no evidence for any antigen-specific differences.

Moreover, a substantial proportion of virus-specific T-cells could cross-react against allo-HLA molecules (54, 55). It has been shown cross-reactivity of CD8+ T cell epitopes between influenza and CMV in mice, and between influenza and EBV in humans (56). Su et al. (57) as well observed that healthy adults can exhibit abundant CD4+ T cells with memory phenotype specific for viral epitopes which those individuals had never encountered, suggesting T cell cross-reactivity as potiential explanation. In our study, clonal CMV-CTL, specific to CMV pp65_495-504_ NLVPMVATV, possibly cross-reacts with 4 WT1 epitope sequences. Although they are nonhomologous, these 4 sequences were, to our knowledge, the first putative cross-reactive epitopes identified between CMV and WT1. Moreover, two different CMV-specific T cell clones presented different cross-creactivity towards epitope sequences of WT1, suggesting private specificity of TCR repertoires (58, 59). Our recent data also demonstrated that patients who developed chronic GHVD habored a higher proportion of CMV-CTL with high affinity (60), as high-affinity T-cells for its antigen were alloreactive which could cross-react against off-target antigens including leukemia antigen and possible allo-HLA, the potential anti-leukemia immunity (61–63).

On the other hand, changes in immune system of CMV-infected individuals do not necessarily induce an enhanced immune response. It could lead to competition for more immune resources and the impaired ability to protect the host from pathogens (64, 65). Our results indicate that the pattern of CMV-CTL apparently changed inversely to that of WT1-CTL, and high expression of TIM-3 and PD-1 on CMV-CTL or WT1-CTL seemed to correlate with positive MRD and later leukemia relapse, maybe as the result from type I IFN-induced apoptosis of memory cells early after infections (66–69). Despite immunosuppression in cancer patients induced by the interaction between tumor cells and immune cells (70), it is unclear whether this immunosuppression in patients with cancer is universal or tumour-antigen specific. Following allo-HSCT, the T cell population as a whole initially expresses high levels of PD-1, and these levels decrease towards normal levels 1-2 years after transplantation (71). Simultaneously, CMV‐specific T cells are functional at ‘normal’ levels expressing fewer exhaustion markers after allo-HSCT, regardless of treatments, compared to tumour‐antigen‐reactive T cells from patients with various cancers except glioblastoma multiforme (32, 72). In the current study, although these CMV-CTL in our cohort were still functional in terms of intracellular cytokine production, leukemia cells may induce the expression of activation/exhaustion molecules on CMV-CTL. Hence, CMV-CTL can proliferate greatly in patients with CMV reactivation, but tumor microenvironments can also alter T cell immune responses against CMV. This suggests that dynamic changes of CMV-specific T cells could be a useful predictor for leukemia relapse, considering higher content of CMV-specific T cells than leukemia-specific T cells in peripheral blood after transplantation.

T cells are traditionally characterized as naïve, central memory, effector memory and terminally differentiated phenotypes based on CD45RA and CCR7 expression. Stem cell-like memory T (TSCM) cells were rare among memory T cells which is distinguished from naïve T cells by surface markers including CD95, transcriptional factors, and effectors after stimulation. Naïve CMV-CTL in our results could have TSCM according to previous reports (73–75). Expansion of virus-specific TSCM protocols by Michael Schmueck-Henneresse (74) and us (76) has been established these years. As naïve T-cell itself is very unlikely to produce cytokine, perforin and granzyme B, the expression of CD95, CD122, and CXCR3 and the production of IFN-γ, TNF-α, perforin, and granzyme B would be helpful to better define the subsets. Admittedly, with the advent of sophisticated ways to defining T cell populations including phenotypes, metabolism, transcription, epigenetics, and single-cell resolution, it will be more useful to consider each T cell is unique about its functions and potentials that different from its sisters. More studies are accordingly warranted to investigate naïve CMV-CTL and virus-specific memory stem T cells in transplant recipients.

This study has several limitations. Its small size and the low number of T-cell events to phenotype and characterize at each time point for every patient affected the accuracy. Four groups with limited cases were available in this analysis: leukemia relapse with CMV reactivation (n=5), no leukemia relapse with CMV reactivation (n=11), no leukemia relapse without CMV reactivation (n=6) and relapse without CMV reactivation (n=2). As we were able to study only a very small number of patients with relapse, additional studies are needed including larger number of patients to explore the relationship between CMV infection and leukemia relapse. Although our previous data, in a different cohort, indicated the majority of the CMV-CTL in the recipients were identified from donor origin early after HSCT (18), the present study also did not differentiate the origin of reconstituted CMV-CTL in the patients. Therefore, we cannot distinguish whether CMV-CTL from donors or recipients may influcence differently the leukemia-specific immune responses. We also found a preliminary result by only two CMV-CTL clones *in vitro* assay. Since these T-cell clones genereated in the present study were straightway stimulated with antigens in the absence of professional antigen presenting cells, it seems that the reactivity depends more on tumor-associated antigens than allo-HLA molecules. Further investigations using the CMV Dextramers defining different sources in a larger population would help to investigate the function of CMV-CTL. Direct data on interaction between a large number of CMV-CTL clones and WT1 would give a clearer picture in the context HSCT setting. Potential public and cross-reactive CMV-specific immune cell subsets could also be geared for cellular immunotherapies in WT1+ AML patients.

In conclusion, we found that CMV infection was accompanied by substantial changes in the frequency of CMV-CTL and Eomes/T-bet expression. Moreover, there is potential cross-reactivity between CMV-specific T cells and WT1. As AML relapse after transplantation was associated with dysregulation of pathways that may influence immune function, but not with the acquired relapse-specific mutations in immune-related genes (77), CMV reactivation and CMV-specific T cells impact immune reconstitution in the long-term, as well as the short-term (78–81). Our results support that CMV reactivation, while influencing immune reconstitution of CMV-CTL, may as well affects WT1 specific immune response by increasing T cell activation, thus potentially contributing to the remission/relapse of AML after transplantation. As CMV infection is one of the major complications after allo-HSCT, it will be important to assess its potential beneficial and detrimental immunity in patients with AML.

## Data availability statement

The original contributions presented in the study are included in the article/**Supplementary Material**. Further inquiries can be directed to the corresponding author.

## Ethics statement

The studies involving human participants were reviewed and approved by Stockholm Ethical Committee South 2010/760-31/1. The patients/participants provided their written informed consent to participate in this study.Written informed consent was obtained from the individual(s) for the publication of any potentially identifiable images or data included in this article.

## Author contributions

X-HL performed experiments, analyzed, interpreted the data, and wrote the manuscript. TP provided patient samples, revised and wrote the final draft, and contributed to the analysis. ZL, QM, and AN performed experiments and interpreted data. PL contributed to revising the manuscript and provided scientific input. X-HL is the guarantor of this work and had full access to all the data in the study and takes responsibility for the integrity of the data and the accuracy of the data analysis. All authors contributed to the article and approved the submitted version.

## Supplementary material

Supplementary Figure 1 Gating approach to classifying WT1-specific or CMV-specific CD8+ T cells by Dextramer staining and intracellular IFN-γ/TNF-α production in response to WT1 or CMV peptides. Representative flow cytometric data showing antigen-specific CD8+ T cells in peripheral blood mononuclear cells (PBMCs): Dextramer+ WT1-specific/CMV-specific CD8+ T cells, IFN-γ+ WT1-specific/CMV-specific CD8+ T cells, and TNF-α+ WT1-specific/CMV-specific CD8+ T cells.

Supplementary Figure 2 Gating approach to classifying memory compartment of CD8+ T cells subpopulations. Representative flow cytometric data showing the four subsets of CD8+ T cells in peripheral blood mononuclear cells (PBMCs): CD45RA+CCR7+ (T_Naive_), CD45RA-CCR7+ (T_CM_), CD45RA-CCR7- (T_EM_), and CD45RA+CCR7- (T_EMRA_). T cell immune responses of one patient (Patient 4) were determined longitudinally at 1, 2, 3, 6, and 12 months after allo-HSCT.

Supplementary Figure 3 Data presented show that CD8+ T cells exhibited four individual patterns of expression for Eomes and T-bet: Eomes-T-bet-, Eomes-T-bet+, Eomes+T-bet-, and Eomes+T-bet+. Peripheral blood mononuclear cells (PBMCs) from transplant recipients were stimulated with CMV pp65 or WT1 protein overnight, stained with anti-CD8 and anti-CD4 and assessed for intracellular expression of IFN-γ, TNF-α, Eomes, and T-bet. IFN-γ and/or TNF-α producing cells are shown as an overlay of total CD4/CD8 T cells.

Supplementary Figure 4 The frequency of CMV-CTL or WT1-CTL identified by intracellular IFN-γ/TNF-α production in patients with CMV reactivation was compared to that of patients without CMV reactivation during the first year after allo-HSCT **(A)**. Memory compartment of CD8+ T cells and WT1-CTL **(B)** and exhaustion marker PD-1, CTLA-4, and TIM-3 on CD8+ T cells and WT1-CTL (C) were also compared in terms of CMV reactivation or not. Graphs present mean values ± SD at each time point. Two-way ANOVA was used to detect differences between two groups over time.

Supplementary Figure 5 The frequency of CMV-CTL or WT1-CTL identified by Dextramer staining or intracellular IFN-γ/TNF-α production in patients with leukemia relapse was compared to that of patients without relapse during the first year after allo-HSCT **(A)**. Memory compartment **(B)** and PD-1, CTLA-4, and TIM-3 **(C)** on CD8+ T cells, and CMV-CTL/WT1-CTL were also compared in terms of leukemia relapse or not. Graphs present mean values ± SD at each time point. Two-way ANOVA was used to detect differences between two groups over time.

Supplementary Figure 6 Eomes/T-bet expression on CD8+ T cells and CMV-CTL/ WT1-CTL was compared in terms of leukemia relapse or not. Graphs present mean values ± SD at each time point. Two-way ANOVA was used to detect differences between two groups over time.

Supplementary Figure 7–11 WT1-CTL and CMV-CTL responses in peripheral blood in relation to disease response as measured by MRD and recipients chimerism. Results in 15 individual patients with detectable WT1 before HSCT are shown (S7: Patient 2, Patient 3, Patient 4; S8: Patient 6, Patient 7, Patient 9; S9: Patient 11, Patient 12, Patient 13; S10: Patient 16, Patient 17, Patient 18; S11: Patient 20, Patient 21, Patient 22). The number of months after transplantation is shown on the x-axis. CMV-CTL and WT1-CTL are expressed as percentage of CD8+ T cells in peripheral blood (middle/ low left, y-axis: green square/ red solid circle). PD-1, TIM-3, and CTLA-4 are expressed as percentage of CMV-CTL/ WT1-CTL (middle/ low right, y-axis: blue solid star/ solid black rhombus/ black triangle/). Disease activity is expressed as MRD identified in bone marrow specimens by flow cytometry (up left, y-axis: triangle in dark green) and recipients chimerism (up right, y-axis: blue circle). Times of donor lymphocytes infusion (DLI), CMV reactivation, graft-versus-host disease (GVHD), and leukemia relapse are depicted on each graph.
