# Corrigendum: Different recovery patterns of CMV-specific and WT1-specific T cells in patients with acute myeloid leukemia undergoing allogeneic hematopoietic cell transplantation: impact of CMV infection and leukemia relapse

**DOI:** 10.3389/fimmu.2023.1195142

**Published:** 2023-05-03

**Authors:** Xiao-Hua Luo, Thomas Poiret, Zhenjiang Liu, Qingda Meng, Anurupa Nagchowdhury, Per Ljungman

**Affiliations:** ^1^ Department of Hematology, The First Affiliated Hospital of Chongqing Medical University, Chongqing, China; ^2^ Department of Laboratory Medicine, Karolinska Institutet, Stockholm, Sweden; ^3^ Department of Cellular Therapy and Allogeneic Stem Cell Transplantation, Karolinska University Hospital and Division of Hematology, Stockholm, Sweden; ^4^ Department of Medicine Huddinge, Karolinska Institutet, Stockholm, Sweden

**Keywords:** cytomegalovirus, WT1 = Wilms tumor 1, immune reconstitution, stem cell transplantation, acute myeloid leukemia (AML)

In the published article, there was an error. The original version of this article inadvertently contained a typographical error.

A correction has been made to **Materials and Methods**, *Determination and characterization of CMV-specific and WT1-specific CD8+ T cells by MHC-Dextramer analyses, Paragraph 1*. This sentence previously stated:

“CMV-specific T cells (CMV-CTL) and WT1-specific T cells (WT1-CTL) frequencies were quantified and phenotyped in patients by staining with PE-A*0201 CMV_NLVPMVATV_ Dextramer, PE-A*0201 WT1_SLLFLLFSL_ Dextramer and APC-A*0201 WT1_VLPLTVAEV_ Dextramer (Immunex, Copenhagen, Denmark) (**Supplementary Figure 1**).”

The corrected sentence appears below:

“CMV-specific T cells (CMV-CTL) and WT1-specific T cells (WT1-CTL) frequencies were quantified and phenotyped in patients by staining with PE-A*0201 CMV_NLVPMVATV_ Dextramer, PE-A*0201 WT1_RMFPNAPYL_ Dextramer and APC-A*0201 WT1_SLGEQQYSV_ Dextramer (Immudex, Copenhagen, Denmark) (**Supplementary Figure 1**).”

The authors apologize for this error and state that this does not change the scientific conclusions of the article in any way. The original article has been updated.

