# Supplementary figures and images for: Different recovery patterns of CMV-specific and WT1-specific T cells in patients with acute myeloid leukemia undergoing allogeneic hematopoietic cell transplantation: Impact of CMV infection and leukemia relapse
Source: Front Immunol. 2023 Feb 7;13:1027593. doi: 10.3389/fimmu.2022.1027593 (PMC9941532; doi:10.3389/fimmu.2022.1027593)

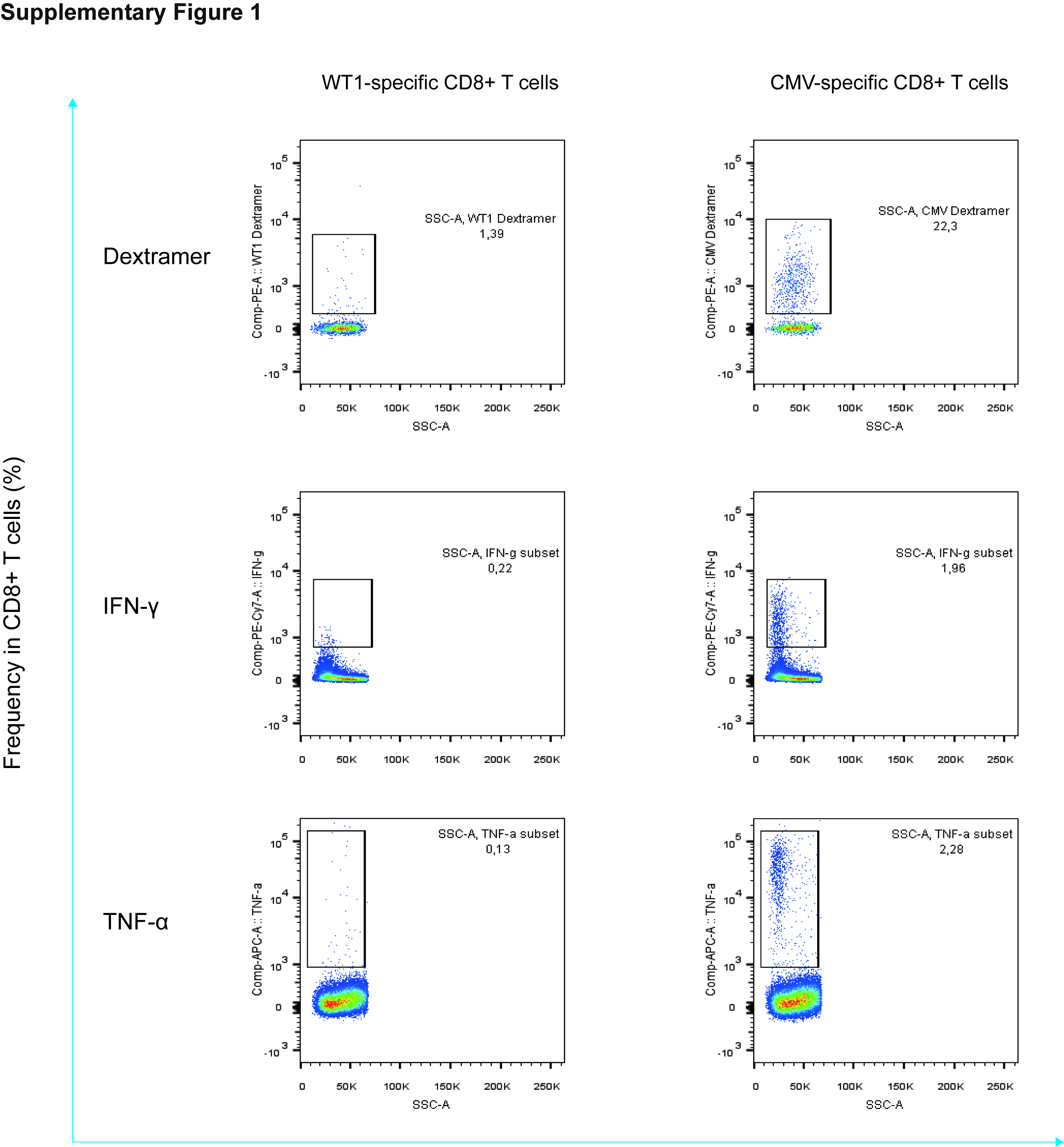

Supplement: Supplementary Figure 1 — Gating approach to classifying WT1-specific or CMV-specific CD8+ T cells by Dextramer staining and intracellular IFN-γ/TNF-α production in response to WT1 or CMV peptides. Representative flow cytometric data showing antigen-specific CD8+ T cells in peripheral blood mononuclear cells (PBMCs): Dextramer+ WT1-specific/CMV-specific CD8+ T cells, IFN-γ+ WT1-specific/CMV-specific CD8+ T cells, and TNF-α+ WT1-specific/CMV-specific CD8+ T cells. [file Image_1.tif]

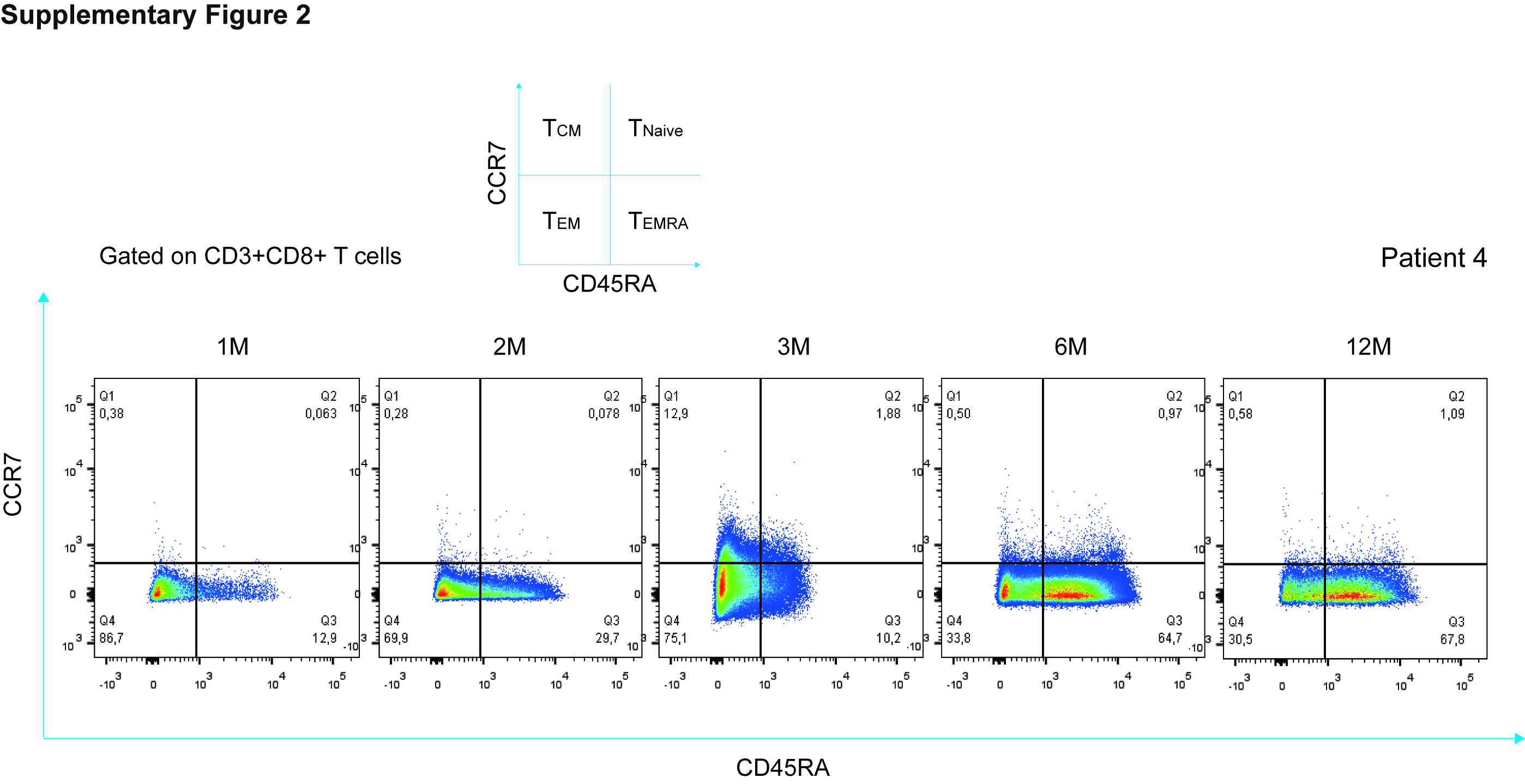

Supplement: Supplementary Figure 2 — Gating approach to classifying memory compartment of CD8+ T cells subpopulations. Representative flow cytometric data showing the four subsets of CD8+ T cells in peripheral blood mononuclear cells (PBMCs): CD45RA+CCR7+ (TNaive), CD45RA-CCR7+ (TCM), CD45RA-CCR7- (TEM), and CD45RA+CCR7- (TEMRA). T cell immune responses of one patient (Patient 4) were determined longitudinally at 1, 2, 3, 6, and 12 months after allo-HSCT. [file Image_2.tif]

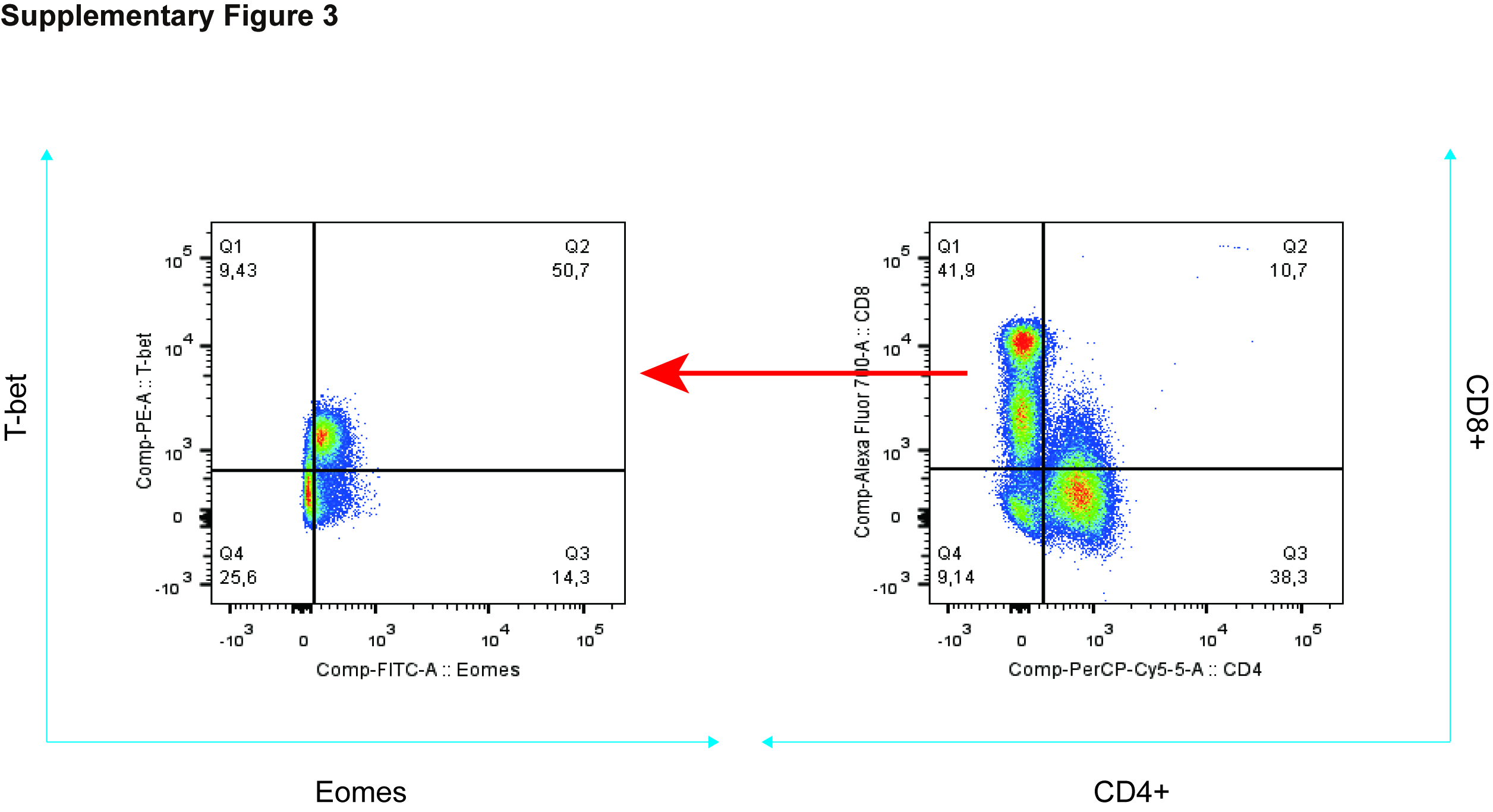

Supplement: Supplementary Figure 3 — Data presented show that CD8+ T cells exhibited four individual patterns of expression for Eomes and T-bet: Eomes-T-bet-, Eomes-T-bet+, Eomes+T-bet-, and Eomes+T-bet+. Peripheral blood mononuclear cells (PBMCs) from transplant recipients were stimulated with CMV pp65 or WT1 protein overnight, stained with anti-CD8 and anti-CD4 and assessed for intracellular expression of IFN-γ, TNF-α, Eomes, and T-bet. IFN-γ and/or TNF-α producing cells are shown as an overlay of total CD4/CD8 T cells. [file Image_3.tif]

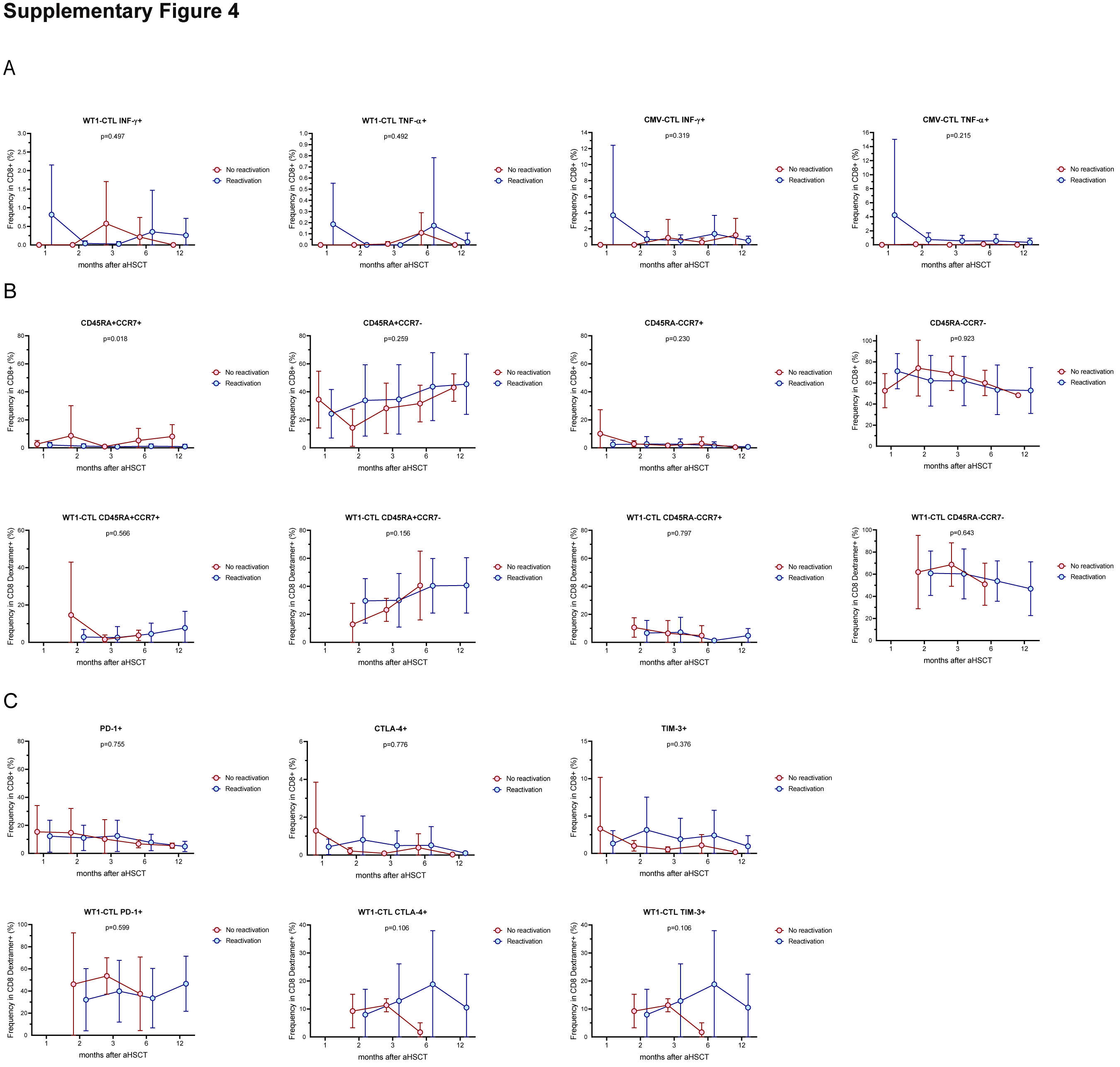

Supplement: Supplementary Figure 4 — The frequency of CMV-CTL or WT1-CTL identified by intracellular IFN-γ/TNF-α production in patients with CMV reactivation was compared to that of patients without CMV reactivation during the first year after allo-HSCT (A). Memory compartment of CD8+ T cells and WT1-CTL (B) and exhaustion marker PD-1, CTLA-4, and TIM-3 on CD8+ T cells and WT1-CTL (C) were also compared in terms of CMV reactivation or not. Graphs present mean values ± SD at each time point. Two-way ANOVA was used to detect differences between two groups over time. [file Image_4.tif]

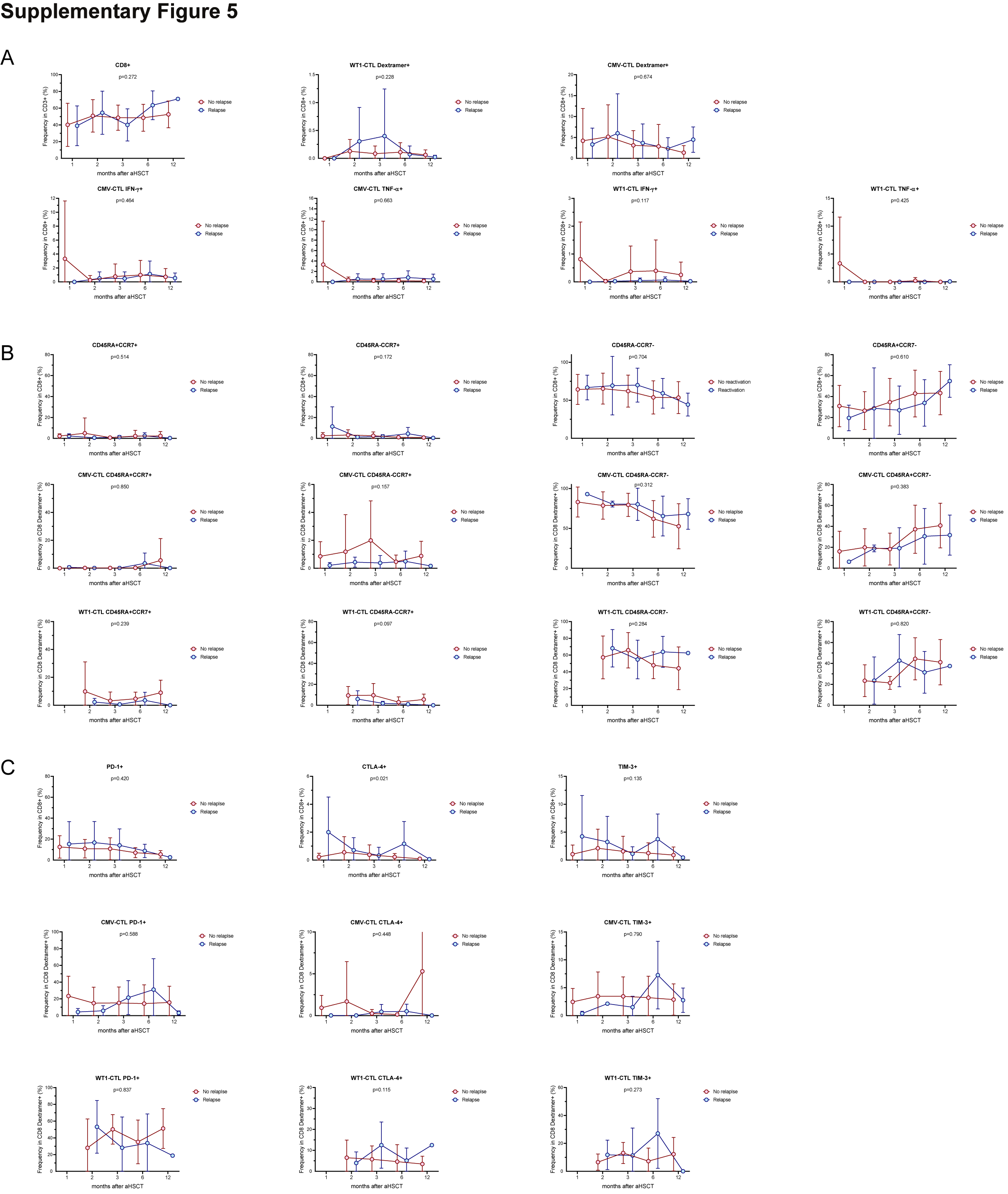

Supplement: Supplementary Figure 5 — The frequency of CMV-CTL or WT1-CTL identified by Dextramer staining or intracellular IFN-γ/TNF-α production in patients with leukemia relapse was compared to that of patients without relapse during the first year after allo-HSCT (A). Memory compartment (B) and PD-1, CTLA-4, and TIM-3 (C) on CD8+ T cells, and CMV-CTL/WT1-CTL were also compared in terms of leukemia relapse or not. Graphs present mean values ± SD at each time point. Two-way ANOVA was used to detect differences between two groups over time. [file Image_5.tif]

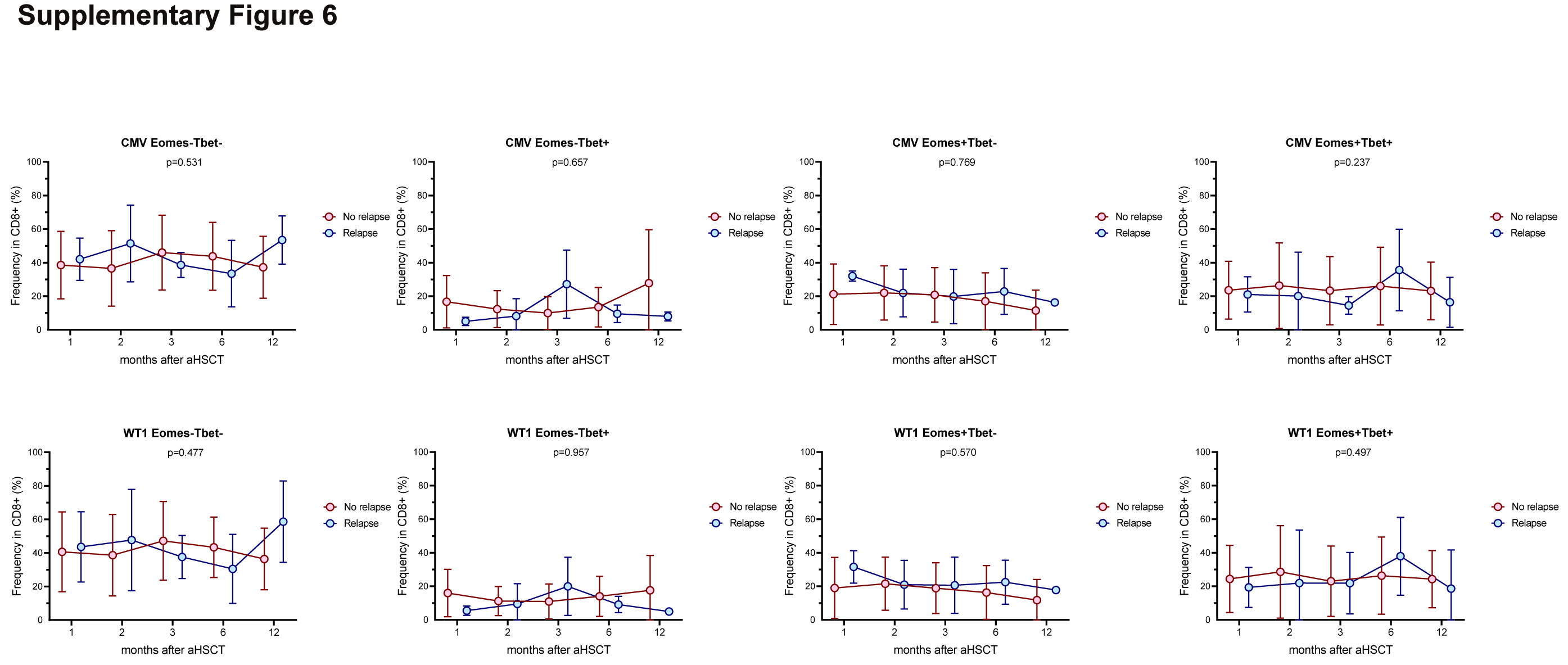

Supplement: Supplementary Figure 6 — Eomes/T-bet expression on CD8+ T cells and CMV-CTL/ WT1-CTL was compared in terms of leukemia relapse or not. Graphs present mean values ± SD at each time point. Two-way ANOVA was used to detect differences between two groups over time. [file Image_6.tif]

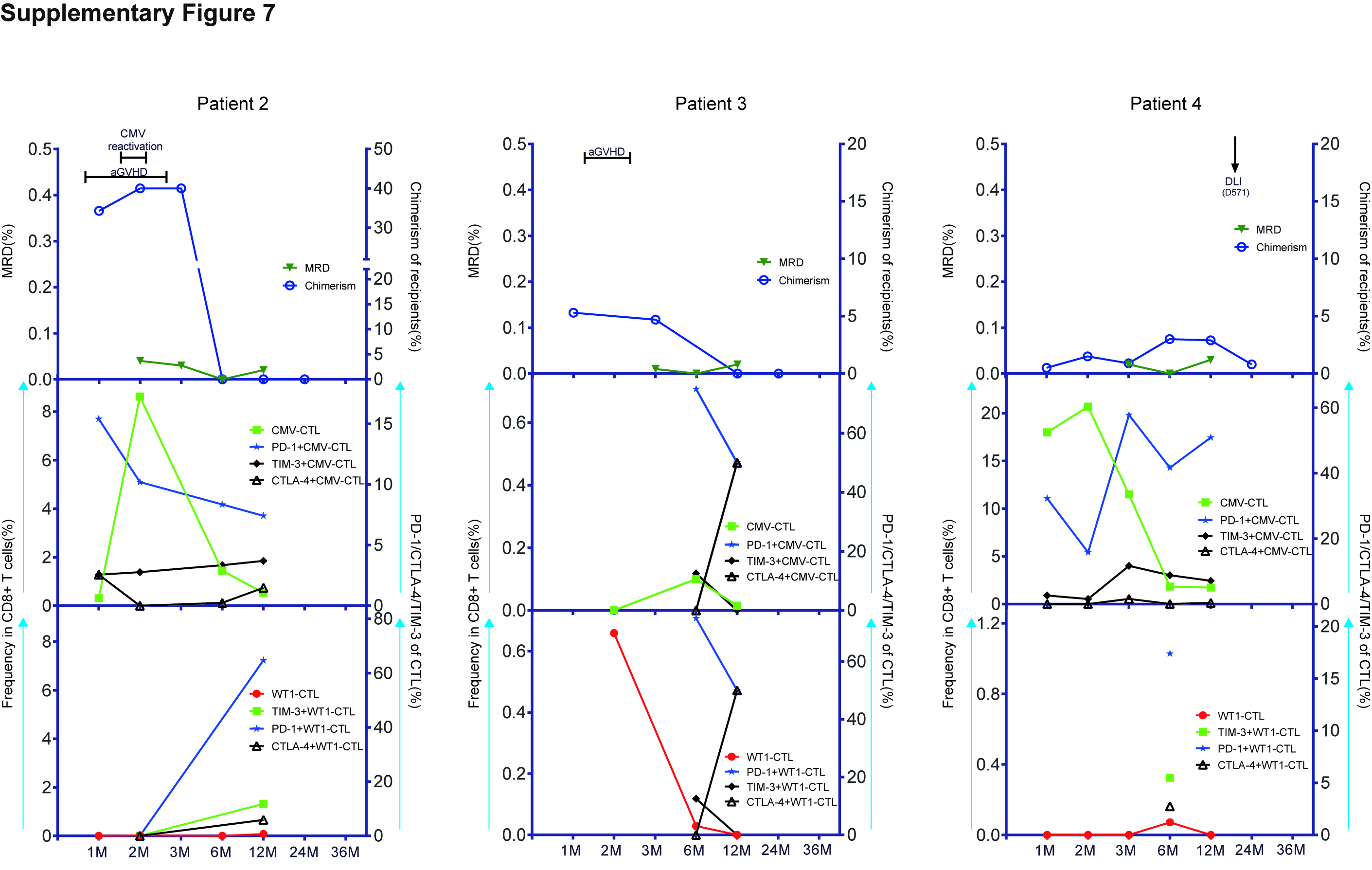

Supplement: Supplementary Figure 7–11 — WT1-CTL and CMV-CTL responses in peripheral blood in relation to disease response as measured by MRD and recipients chimerism. Results in 15 individual patients with detectable WT1 before HSCT are shown (S7: Patient 2, Patient 3, Patient 4; S8: Patient 6, Patient 7, Patient 9; S9: Patient 11, Patient 12, Patient 13; S10: Patient 16, Patient 17, Patient 18; S11: Patient 20, Patient 21, Patient 22). The number of months after transplantation is shown on the x-axis. CMV-CTL and WT1-CTL are expressed as percentage of CD8+ T cells in peripheral blood (middle/ low left, y-axis: green square/ red solid circle). PD-1, TIM-3, and CTLA-4 are expressed as percentage of CMV-CTL/ WT1-CTL (middle/ low right, y-axis: blue solid star/ solid black rhombus/ black triangle/). Disease activity is expressed as MRD identified in bone marrow specimens by flow cytometry (up left, y-axis: triangle in dark green) and recipients chimerism (up right, y-axis: blue circle). Times of donor lymphocytes infusion (DLI), CMV reactivation, graft-versus-host disease (GVHD), and leukemia relapse are depicted on each graph. [file Image_7.tif]

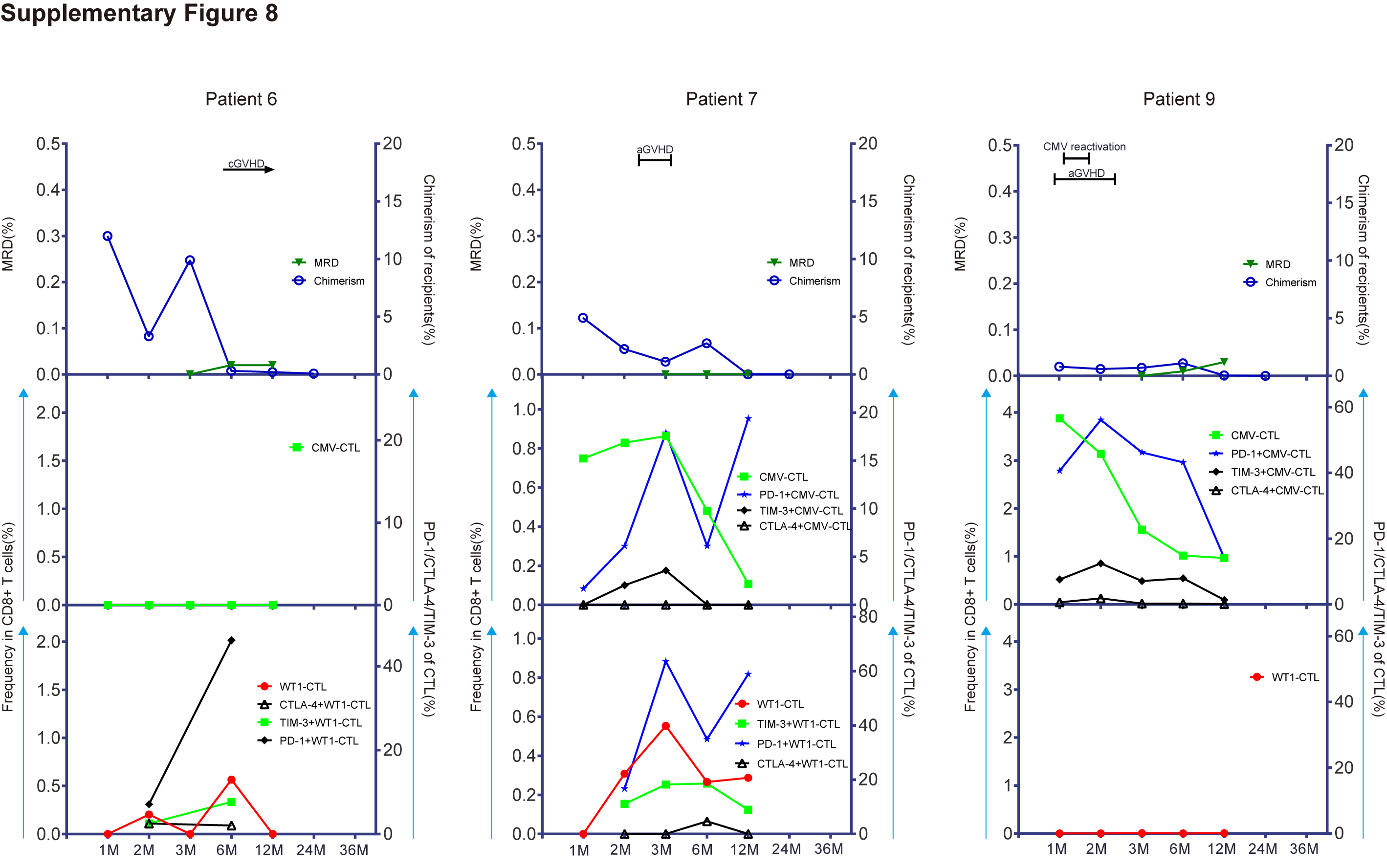

Supplement: Supplementary file 8 [file Image_8.tif]

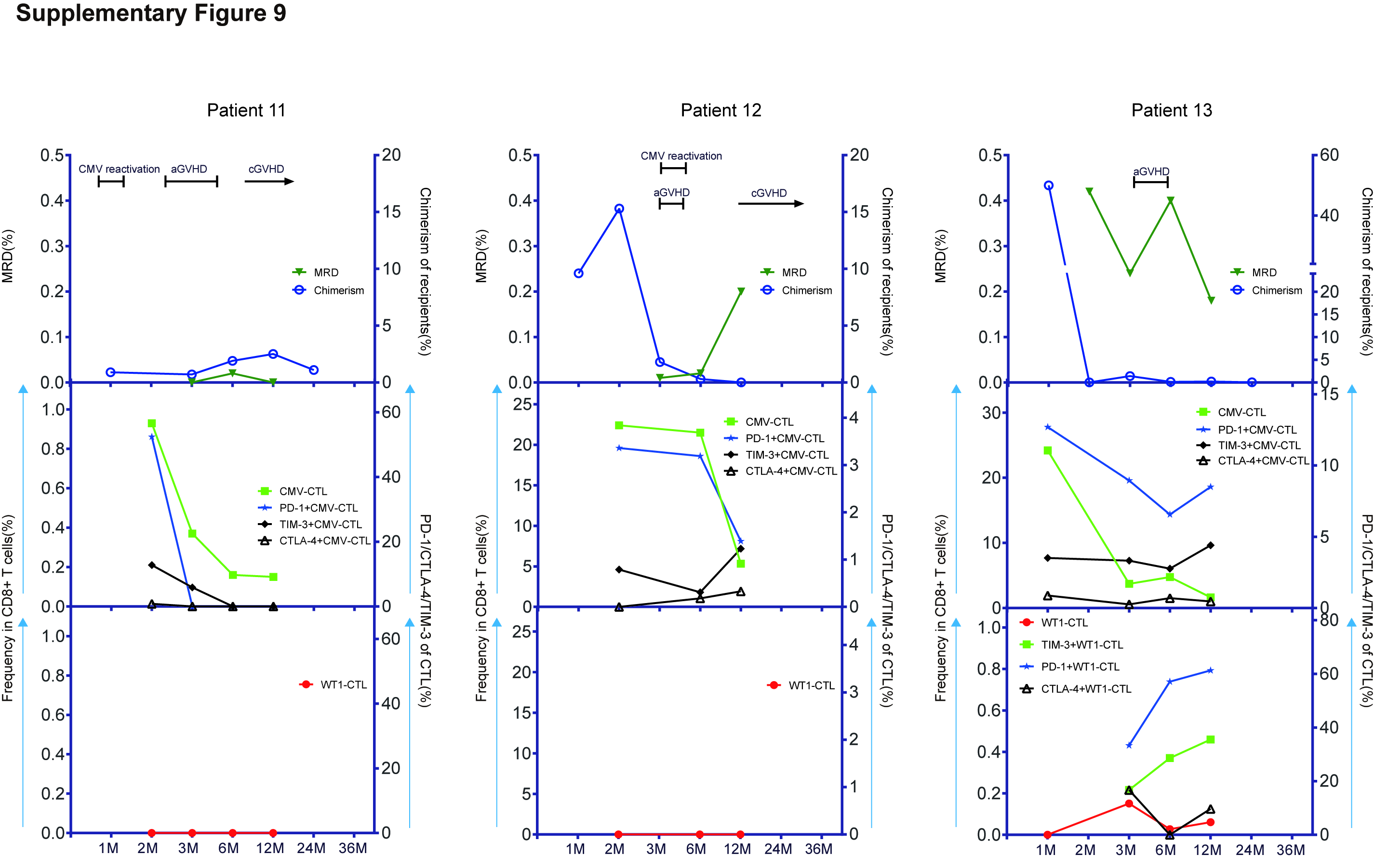

Supplement: Supplementary file 9 [file Image_9.tif]

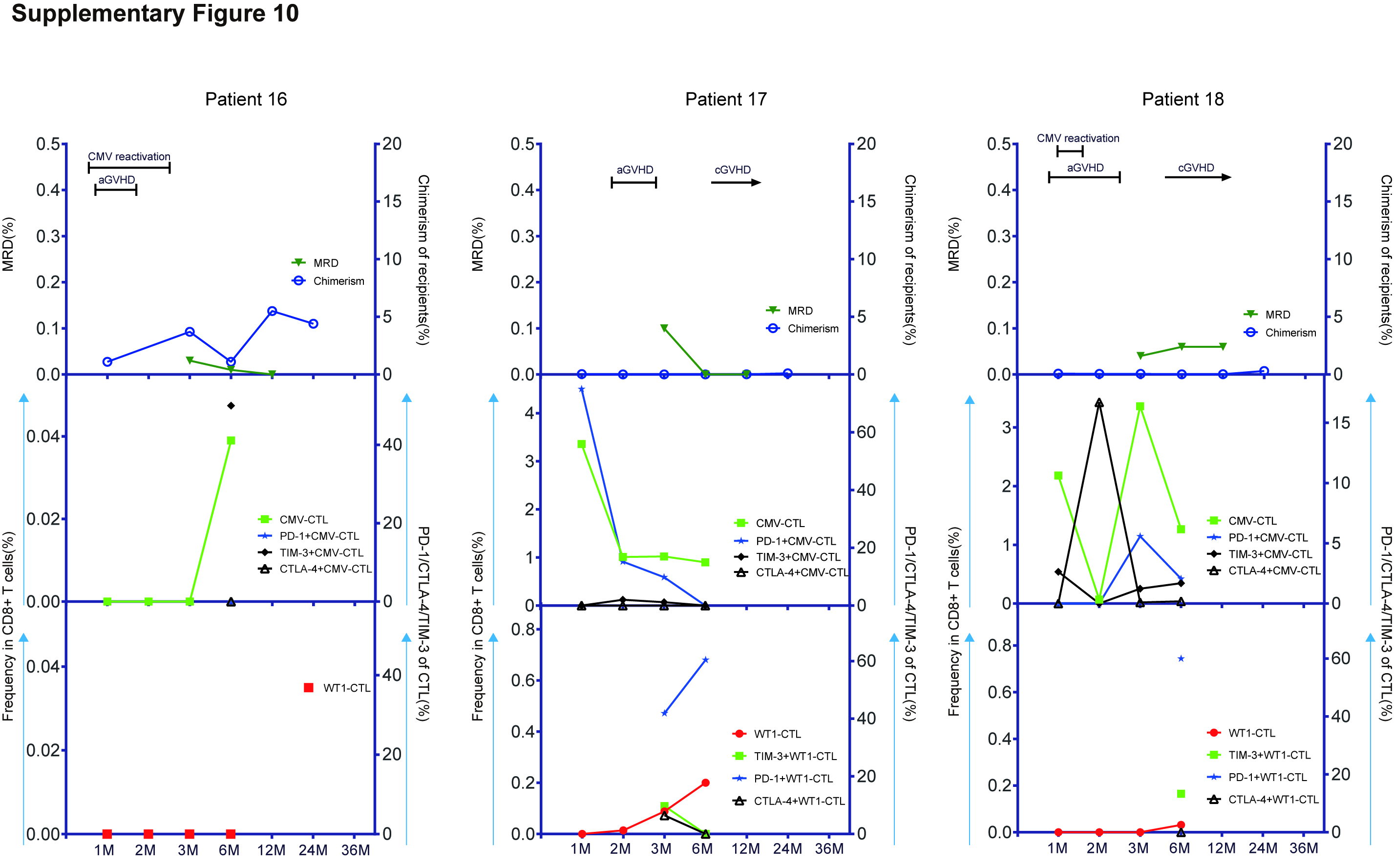

Supplement: Supplementary file 10 [file Image_10.tif]

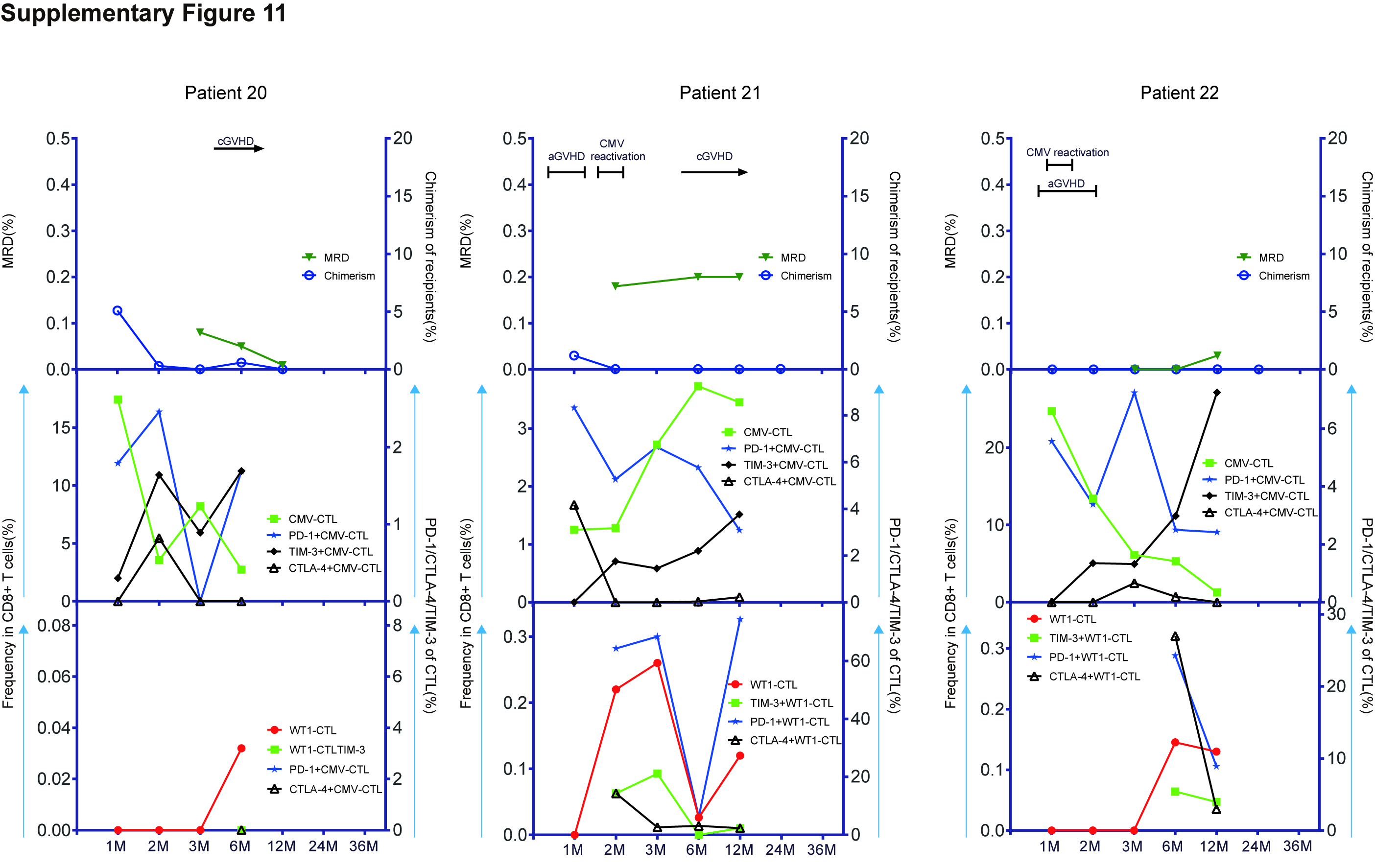

Supplement: Supplementary file 11 [file Image_11.tif]
