# Supplementary material for: Different recovery patterns of CMV-specific and WT1-specific T cells in patients with acute myeloid leukemia undergoing allogeneic hematopoietic cell transplantation: Impact of CMV infection and leukemia relapse
Source: Front Immunol. 2023 Feb 7;13:1027593. doi: 10.3389/fimmu.2022.1027593 (PMC9941532; doi:10.3389/fimmu.2022.1027593)
Supplement: Supplementary file 12 [file DataSheet_1.zip › Supplementary table.docx]

S1 The availability of samples per donor for each time point during the follow-up

| **ID** | **1M** | **2M** | **3M** | **6M** | **12M** |
| --- | --- | --- | --- | --- | --- |
|  | Methods | Methods | Methods | Methods | Methods |
| **1** |  | P+ICS |  | P+ICS |  |
| **2** | P+ICS | P+ICS |  | P+ICS | P |
| **3** |  | P |  | P | P+ICS |
| **4** | P+ICS | P+ICS | P | P+ICS | P+ICS |
| **5** | P | P+ICS | P+ICS | P+ICS |  |
| **6** | P+ICS | P+ICS | P+ICS | P+ICS | P+ICS |
| **7** | P | P | P | P+ICS | P+ICS |
| **8** | P | P+ICS | P+ICS |  |  |
| **9** | P+ICS | P | P+ICS | P+ICS | P+ICS |
| **10** |  |  | P | P | P+ICS |
| **11** |  | P+ICS | P+ICS | P+ICS | P+ICS |
| **12** |  | P |  | P | P+ICS |
| **13** | P+ICS |  | P+ICS | P+ICS | P+ICS |
| **14** | P+ICS | P+ICS | P+ICS | P+ICS | P+ICS |
| **15** | ICS | ICS | ICS | ICS | ICS |
| **16** | P+ICS | P+ICS | P+ICS | P+ICS |  |
| **17** | P | P | P+ICS | P+ICS |  |
| **18** | P | P | P+ICS | P+ICS |  |
| **19** | P+ICS | P+ICS | P+ICS | P+ICS |  |
| **20** | P | P+ICS | P+ICS | P+ICS |  |
| **21** | P+ICS | P | P+ICS | P | P |
| **22** | P+ICS | P | P | P+ICS | P+ICS |
| **23** | P+ICS | P+ICS | P+ICS | P+ICS |  |
| **24** | P+ICS |  |  | P+ICS |  |

P, Phenotyping CMV-specific and WT1-specific CD8+ T cells by MHC-dextramer and/or surface antibodies staining; ICS, Identifying CD8+ or CD4+ T cells producing IFN-γ/TNF-α and the level of Eomes/ T-bet in response to secondary stimulation with the CMV pp65 protein or WT1 protein.

S2 Sequences of WT1 peptides

| Name | N-term | sequence | C-term | length |
| --- | --- | --- | --- | --- |
| WT1_2 | H | VRDLNALLPAVPSLG | NH2 | 15 |
| WT1_3 | H | NALLPAVPSLGGGGG | NH2 | 15 |
| WT1_4 | H | PAVPSLGGGGGCALP | NH2 | 15 |
| WT1_5 | H | SLGGGGGCALPVSGA | NH2 | 15 |
| WT1_6 | H | GGGCALPVSGAAQWA | NH2 | 15 |
| WT1_7 | H | ALPVSGAAQWAPVLD | NH2 | 15 |
| WT1_8 | H | SGAAQWAPVLDFAPP | NH2 | 15 |
| WT1_9 | H | QWAPVLDFAPPGASA | NH2 | 15 |
| WT1_10 | H | VLDFAPPGASAYGSL | NH2 | 15 |
| WT1_11 | H | APPGASAYGSLGGPA | NH2 | 15 |
| WT1_12 | H | ASAYGSLGGPAPPPA | NH2 | 15 |
| WT1_13 | H | GSLGGPAPPPAPPPP | NH2 | 15 |
| WT1_14 | H | GPAPPPAPPPPPPPP | NH2 | 15 |
| WT1_15 | H | PPAPPPPPPPPPHSF | NH2 | 15 |
| WT1_16 | H | PPPPPPPPHSFIKQE | NH2 | 15 |
| WT1_17 | H | PPPPHSFIKQEPSWG | NH2 | 15 |
| WT1_18 | H | HSFIKQEPSWGGAEP | NH2 | 15 |
| WT1_19 | H | KQEPSWGGAEPHEEQ | NH2 | 15 |
| WT1_20 | H | SWGGAEPHEEQCLSA | NH2 | 15 |
| WT1_21 | H | AEPHEEQCLSAFTVH | NH2 | 15 |
| WT1_22 | H | EEQCLSAFTVHFSGQ | NH2 | 15 |
| WT1_23 | H | LSAFTVHFSGQFTGT | NH2 | 15 |
| WT1_24 | H | TVHFSGQFTGTAGAC | NH2 | 15 |
| WT1_25 | H | SGQFTGTAGACRYGP | NH2 | 15 |
| WT1_26 | H | TGTAGACRYGPFGPP | NH2 | 15 |
| WT1_27 | H | GACRYGPFGPPPPSQ | NH2 | 15 |
| WT1_28 | H | YGPFGPPPPSQASSG | NH2 | 15 |
| WT1_29 | H | GPPPPSQASSGQARM | NH2 | 15 |
| WT1_30 | H | PSQASSGQARMFPNA | NH2 | 15 |
| WT1_31 | H | SSGQARMFPNAPYLP | NH2 | 15 |
| WT1_32 | H | ARMFPNAPYLPSCLE | NH2 | 15 |
| WT1_33 | H | PNAPYLPSCLESQPA | NH2 | 15 |
| WT1_34 | H | YLPSCLESQPAIRNQ | NH2 | 15 |
| WT1_35 | H | CLESQPAIRNQGYST | NH2 | 15 |
| WT1_36 | H | QPAIRNQGYSTVTFD | NH2 | 15 |
| WT1_37 | H | RNQGYSTVTFDGTPS | NH2 | 15 |
| WT1_38 | H | YSTVTFDGTPSYGHT | NH2 | 15 |
| WT1_39 | H | TFDGTPSYGHTPSHH | NH2 | 15 |
| WT1_40 | H | TPSYGHTPSHHAAQF | NH2 | 15 |
| WT1_41 | H | GHTPSHHAAQFPNHS | NH2 | 15 |
| WT1_42 | H | SHHAAQFPNHSFKHE | NH2 | 15 |
| WT1_43 | H | AQFPNHSFKHEDPMG | NH2 | 15 |
| WT1_44 | H | NHSFKHEDPMGQQGS | NH2 | 15 |
| WT1_45 | H | KHEDPMGQQGSLGEQ | NH2 | 15 |
| WT1_46 | H | PMGQQGSLGEQQYSV | NH2 | 15 |
| WT1_47 | H | QGSLGEQQYSVPPPV | NH2 | 15 |
| WT1_48 | H | GEQQYSVPPPVYGCH | NH2 | 15 |
| WT1_49 | H | YSVPPPVYGCHTPTD | NH2 | 15 |
| WT1_50 | H | PPVYGCHTPTDSCTG | NH2 | 15 |
| WT1_51 | H | GCHTPTDSCTGSQAL | NH2 | 15 |
| WT1_52 | H | PTDSCTGSQALLLRT | NH2 | 15 |
| WT1_53 | H | CTGSQALLLRTPYSS | NH2 | 15 |
| WT1_54 | H | QALLLRTPYSSDNLY | NH2 | 15 |
| WT1_55 | H | LRTPYSSDNLYQMTS | NH2 | 15 |
| WT1_56 | H | YSSDNLYQMTSQLEC | NH2 | 15 |
| WT1_57 | H | NLYQMTSQLECMTWN | NH2 | 15 |
| WT1_58 | H | MTSQLECMTWNQMNL | NH2 | 15 |
| WT1_59 | H | LECMTWNQMNLGATL | NH2 | 15 |
| WT1_60 | H | TWNQMNLGATLKGVA | NH2 | 15 |
| WT1_61 | H | MNLGATLKGVAAGSS | NH2 | 15 |
| WT1_62 | H | ATLKGVAAGSSSSVK | NH2 | 15 |
| WT1_63 | H | GVAAGSSSSVKWTEG | NH2 | 15 |
| WT1_64 | H | GSSSSVKWTEGQSNH | NH2 | 15 |
| WT1_65 | H | SVKWTEGQSNHSTGY | NH2 | 15 |
| WT1_66 | H | TEGQSNHSTGYESDN | NH2 | 15 |
| WT1_67 | H | SNHSTGYESDNHTTP | NH2 | 15 |
| WT1_68 | H | TGYESDNHTTPILCG | NH2 | 15 |
| WT1_69 | H | SDNHTTPILCGAQYR | NH2 | 15 |
| WT1_70 | H | TTPILCGAQYRIHTH | NH2 | 15 |
| WT1_71 | H | LCGAQYRIHTHGVFR | NH2 | 15 |
| WT1_72 | H | QYRIHTHGVFRGIQD | NH2 | 15 |
| WT1_73 | H | HTHGVFRGIQDVRRV | NH2 | 15 |
| WT1_74 | H | VFRGIQDVRRVPGVA | NH2 | 15 |
| WT1_75 | H | IQDVRRVPGVAPTLV | NH2 | 15 |
| WT1_76 | H | RRVPGVAPTLVRSAS | NH2 | 15 |
| WT1_77 | H | GVAPTLVRSASETSE | NH2 | 15 |
| WT1_78 | H | TLVRSASETSEKRPF | NH2 | 15 |
| WT1_79 | H | SASETSEKRPFMCAY | NH2 | 15 |
| WT1_80 | H | TSEKRPFMCAYPGCN | NH2 | 15 |
| WT1_81 | H | RPFMCAYPGCNKRYF | NH2 | 15 |
| WT1_82 | H | CAYPGCNKRYFKLSH | NH2 | 15 |
| WT1_83 | H | GCNKRYFKLSHLQMH | NH2 | 15 |
| WT1_84 | H | RYFKLSHLQMHSRKH | NH2 | 15 |
| WT1_85 | H | LSHLQMHSRKHTGEK | NH2 | 15 |
| WT1_86 | H | QMHSRKHTGEKPYQC | NH2 | 15 |
| WT1_87 | H | RKHTGEKPYQCDFKD | NH2 | 15 |
| WT1_88 | H | GEKPYQCDFKDCERR | NH2 | 15 |
| WT1_89 | H | YQCDFKDCERRFSRS | NH2 | 15 |
| WT1_90 | H | FKDCERRFSRSDQLK | NH2 | 15 |
| WT1_91 | H | ERRFSRSDQLKRHQR | NH2 | 15 |
| WT1_92 | H | SRSDQLKRHQRRHTG | NH2 | 15 |
| WT1_93 | H | QLKRHQRRHTGVKPF | NH2 | 15 |
| WT1_94 | H | HQRRHTGVKPFQCKT | NH2 | 15 |
| WT1_95 | H | HTGVKPFQCKTCQRK | NH2 | 15 |
| WT1_96 | H | KPFQCKTCQRKFSRS | NH2 | 15 |
| WT1_97 | H | CKTCQRKFSRSDHLK | NH2 | 15 |
| WT1_98 | H | QRKFSRSDHLKTHTR | NH2 | 15 |
| WT1_99 | H | SRSDHLKTHTRTHTG | NH2 | 15 |
| WT1_100 | H | HLKTHTRTHTGKTSE | NH2 | 15 |
| WT1_101 | H | HTRTHTGKTSEKPFS | NH2 | 15 |
| WT1_102 | H | HTGKTSEKPFSCRWP | NH2 | 15 |
| WT1_103 | H | TSEKPFSCRWPSCQK | NH2 | 15 |
| WT1_104 | H | PFSCRWPSCQKKFAR | NH2 | 15 |
| WT1_105 | H | RWPSCQKKFARSDEL | NH2 | 15 |
| WT1_106 | H | CQKKFARSDELVRHH | NH2 | 15 |
| WT1_107 | H | FARSDELVRHHNMHQ | NH2 | 15 |
| WT1_108 | H | DELVRHHNMHQRNMT | NH2 | 15 |
| WT1_109 | H | RHHNMHQRNMTKLQL | NH2 | 15 |
| WT1_110 | H | HNMHQRNMTKLQLAL | NH2 | 15 |
